# Carveoylphenols and Their Antifungal Potential against Pathogenic Yeasts

**DOI:** 10.3390/antibiotics8040185

**Published:** 2019-10-15

**Authors:** Iván Montenegro, Marco Mellado, Alessandra Russo, Bastian Said, Ximena Besoain, Patricio Godoy, Enrique Werner, Nelson Caro, Alejandro Madrid

**Affiliations:** 1 Escuela de Obstetricia y Puericultura, Facultad de medicina, Universidad de Valparaíso, Angamos 655, Reñaca, Viña del Mar 2520000, Chile; ivan.montenegro@uv.cl; 2 Instituto de Química, Facultad de Ciencias, Pontificia Universidad Católica de Valparaíso, Av. Universidad #330, Curauma, Valparaíso 2340000, Chile; marco.mellado@pucv.cl; 3 Department of Drug Sciences, University of Catania, Via S. Sofia 64, 95125 Catania, Italy; alrusso@unict.it; 4 Departamento de Química, Universidad Técnica Federico Santa María, Av. Santa María 6400, Vitacura 7630000, Santiago, Chile; bastian.said@usm.cl; 5 Escuela de Agronomía Pontificia Universidad Católica de Valparaíso, Quillota, SanFrancisco s/n La Palma, Quillota 2260000, Chile; ximena.besoain@pucv.cl; 6 Instituto de Microbiología Clínica, Facultad de Medicina, Universidad Austral de Chile, Los Laureles s/n, Isla Teja, Valdivia 5090000, Chile; patricio.godoy@uach.cl; 7 Departamento de Ciencias Básicas, Campus Fernando May, Universidad del Bío-Bío, Avda. Andrés Bello 720, casilla 447, Chillán 3780000, Chile; ewerner@ubiobio.cl; 8 Centro de Investigación Australbiotech, Universidad Santo Tomás, Avda. Ejército 146, Santiago 8320000, Chile; ncaro@australbiotech.cl; 9 Laboratorio de Productos Naturales y Síntesis Orgánica (LPNSO), Departamento de Química, Facultad de Ciencias Naturales y Exactas, Universidad de Playa Ancha, Avda. Leopoldo Carvallo 270, Playa Ancha, Valparaíso 2340000, Chile

**Keywords:** carveol, carveoylphenols, titanium tetrachloride, antifungal, *Candida* sp.

## Abstract

*Candida* is a genus of yeasts and is the most common cause of fungal infections worldwide. However, only a few antifungal drugs are currently available for the treatment of *Candida* infections. In the last decade, terpenophenols have attracted much attention because they often possess a variety of biological activities. In the search for new antifungals, eight carveoylphenols were synthesized and characterized by spectroscopic analysis. By using the broth microdilution assay, the compounds were evaluated for antifungal activities in vitro against four human pathogenic yeast, and structure–activity relationships (SAR) were derived. Noteworthy, in this preliminary study, compounds **5** and **6**, have shown a significant reduction in the growth of all *Candida* strains tested. Starting from these preliminary results, we have designed the second generation of analogous in this class, and further studies are in progress in our laboratories.

## 1. Introduction

In recent decades, fungal infections have gained greater medical importance, given the sustained increase in new infections usually caused by opportunistic fungi, with the *Candida* genus being one of the most important. Candidiasis is a sporadic and or chronic infection caused by different yeasts of the genus *Candida* sp., a type of fungus that is common in the body of healthy people. The pathology presents with extremely variable clinical manifestations of acute, subacute, chronic, or episodic evolution, in which the fungus can cause cutaneous and mucocutaneous lesions. However, in patients with various underlying diseases or host factors, *Candida* may cause invasive disease (invasive candidiasis or candidosis), most often as bloodstream infection (candidaemia) with or without secondary dissemination to the eyes, liver, spleen, bones, heart valves, central nervous system and so on or as deep-seated candidiasis, such as peritonitis after gastrointestinal surgery [1]. Although more than 17 pathogenic species have been reported, 90% of infections are attributed to *Candida albicans*, *C. glabrata*, *C. krusei*, *C. parasilopsis,* and *C. tropicalis* [2].

For many years, *C. albicans* was regarded as the main cause of invasive fungal infections, but lately, non-*C. albicans* have been reported to be predominant, especially in hospital environments [3].

In the last decade, there has been a significant increase in non-albicans *Candida* species, as causative agents of opportunistic fungal infections, including *C. glabrata*, *C. lusitaniae,* and *C. guilliermondii* [4]. *C. glabrata* may be the second most common *Candida* strain, with its prevalence growing since the 1990s [2,3,4]. *C. lusitaniae* is a rare opportunistic yeast that is known for its resistance to amphotericin B (AmB). It is responsible for about 19.3% of all infections caused by non-*C. albicans* species, and for about 1.7% of all cases of genitourinary candidiasis brought about by the entire spectrum of the *Candida* species [5]. In South America, 10.2% of isolates of *C. guilliermondii* resistant to fluconazole have been reported, with 12.9% of sensitive isolations dependent on the dose [6].

Genomic and proteomic studies have been carried out with *C. albicans*, *C. glabrata* [7], *C. lusitanie* [8], and *C. krusei* [9] to determine the molecular mechanisms that govern the resistance to fluconazole. Genes, proteins, and metabolic pathways associated with these processes have been identified [10,11,12,13].

*Candida* infected patients are commonly treated worldwide with a variety of antifungal drugs such as fluconazole, amphotericin B, nystatin, and flucytosine. Moreover, early detection and speciation of the fungal agents will play a crucial role in administering appropriate drugs for antifungal therapy [14].

Currently, there are several families of antifungals available in the market, with azoles being one of the most common, which inhibit the enzyme 14 α-lanosterol-demethylase, affecting the biosynthesis of ergosterol, an important component of the fungal plasma membrane [15].

Polyene antifungals such as amphotericin B act by binding to ergosterol in the fungal cell membrane. This binding results in depolarization of the membrane and formation of pores that increase permeability to proteins and monovalent and divalent cations, eventually leading to cell death [16].

In Chile, strains with decreased susceptibility to azoles have been found, especially in outpatients, probably associated with the frequent use of antifungals for the treatment of superficial infections, such as vulvovaginal candidiasis [17]. Molecular mechanisms described in the azole resistance are: Overexpression of efflux pumps (transmembrane proteins of the antiport type encoded by the multidrug resistance (MDR) or complementarity determining region (CDR) genes), which uses a proton gradient (H+) to translocate molecules does not allow the accumulation of the drug in the intracellular compartment [18]. Probably one of the most important mechanisms of resistance to azoles is the failure of the intracellular accumulation of the drug by overexpression of the genes encoding the pumps. The heterologous expression of the CDR1 and CDR2 genes in *Saccharomyces cerevisiae* confers resistance to several azoles (fluconazole, itraconazole, and ketoconazole) and to other antifungals and metabolic inhibitors [19].

As per the literature, previous studies have demonstrated that natural products have effects as antibiofilm, antiviral, antifungal, antimicrobial, angioedema, analgesic, food packing, biodegradable films, and antioxidant activities [20]. For example, volatile oil of *Trachyspermum ammi* contains monoterpenes (thymol, γ-terpinene, *p*-cymene, carveol, and β-pinene) [21]. Carveol (**1**) is an oil that is available commercially. It is recognized as an anti-bacterial agent, but heretofore, had not been recognized to have anti-yeast or antifungal activity [22]. The exact method of killing fungi is unknown, but it is thought that carveol (**1**) kills fungi by lysing the cell membrane of the organism, which is lethal to the organism. In this sense, the use of monoterpenes such as carveol (**1**) represents an important source to obtain antifungal compounds with a broad spectrum of action. Aiming at a possible increase in the efficiency of the biological action of carveol, we have performed chemical modification via the introduction of phenol groups. However, there are scarce data in the literature concerning the antifungal activity of these types of molecules, such as monoterpenophenols.

**Figure 1:**
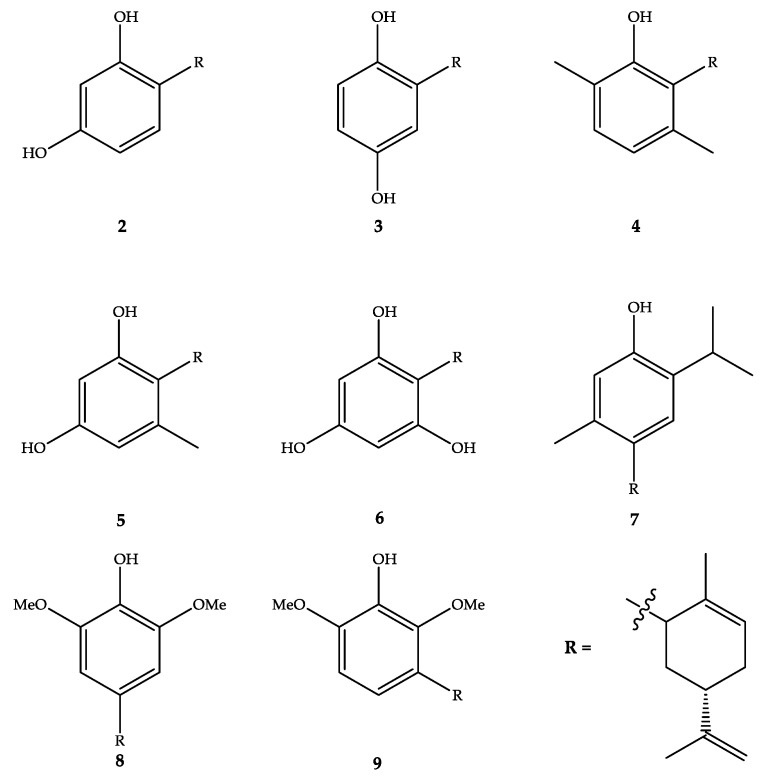
Structure of the carveoylphenols.

A series of eight carveoylphenols (**2**–**9**, Figure 1) were designed, synthesized, and evaluated for their antifungal activity against four strains of *Candida* sp. The results are discussed in terms of the structural features of the tested compounds, trying to establish a structure–activity relationship.

## 2. Results

### 2.1. Synthesis of Carveoylphenols

**Scheme 1:**
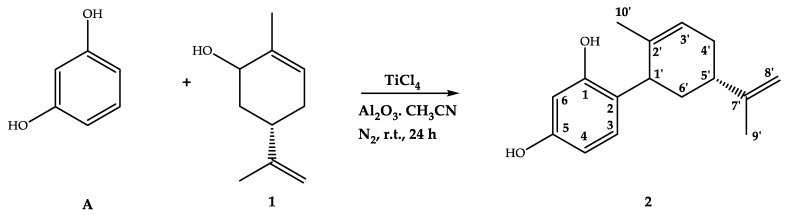
Reaction scheme for carveoylphenol **2** synthesis.

The synthesis of terpenphenols has experienced explosive growth in recent years because they exhibit a variety of biological activities, both in vivo and in vitro models [23]. A large quantity of the literature is dedicated to the synthesis protocols. The methodology is based on the typical alkylation of phenols by monoterpenoid allylic alcohols with variation of the type and activity of the catalysts [24]. However, cyclic monoterpen allylic alcohols exhibit fewer examples of reactions with phenols [24]. We recently reported the synthesis of cyclodiprenyl phenols from perillyl alcohol by alkylation with the corresponding phenol in the presence of boron trifluoride diethyl etherate as a catalyst with acetonitrile as a solvent [25]. In this work, the direct coupling of carveol (**1**) to different phenols (resorcinol (**A**), hydroquinone (**B**), 2,5-dimethylphenol (**C**), orcinol (**D**), phloroglucinol (**E**), thymol (**F**) and 2,6-dimethoxyphenol (**G**) (Supplementary Material, Figure S1)) was performed in a suspension of aluminum oxide in acetonitrile using titanium tetrachloride as a catalyst, and the time reaction was reduced to 24 h (Scheme 1).

Carveoylphenols **2**–**9** were obtained in moderate to good yields (20.0–89.0%), and the chemical structures were elucidated via IR, ^1^H-NMR, ^13^C-NMR and HRMS spectroscopic methods (Supplementary Material, Spectra S1).

The linkage occurred mainly between carbon 1’ of the carveol moiety and carbon 2 of the phenols. It is of interest to point out that, by contrast, while alkylation of monoterpenes like thymol and carvacrol [26] takes place mostly at the C-4 position, alkylation of the other phenols take place preferentially at the C-2 position.

The alkylation reaction using boron trifluoride etherate between dimethoxyphenols and monoterpenoid allylic alcohols in acetonitrile did not show a coupling reaction [27]. By contrast, under these conditions the condensation of 2,6-dimethoxyphenol (**G**) with 1 gave two compounds; **8** as the major product in a 48.8% yield and **9** as the minor product in a 20.0% yield

### 2.2. Antifungal Activity

The in vitro antifungal activities of carveol (**1**) and all the synthesized derivatives **2**–**9** were screened at between 0.03–16.0 µg/mL concentrations using various Candida strains, including *C. glabrata*, *C. lusitaniae*, *C. guilliermondii*, and *C. albicans* following the protocol of the National Committee for Clinical Laboratory Standards (NCCLS) [28]. Itraconazole and ketoconazole were selected as positive controls. The results of the antifungal potential of the compounds presented in Table 1 and Table 2 indicated that among all the strains, *C. lusitaniae* was the most susceptible to synthesized compounds.

Compounds **5** and **6** showed a high value of MIC for all the strains, independent of the time of exposure to the drug. The activities presented by both compounds were found to be comparable or superior to the positive controls. At the same time, compound **7** showed great antifungal activity, except for *C. glabrata*. However, compound **6** was the most potent derivative of the series, with MIC_80_ values at 48 h of 1.0 µg/mL, 0.50 µg/mL, 1.0 µg/mL, and 1.0 µg/mL against *C. glabrata*, *C. lusitaniae*, *C. guilliermondii*, and *C. albicans*, respectively. While itraconazole showed antifungal activity with MIC_80_ values at 48 h of >16 µg/mL, 4.0 µg/mL, 8.0 µg/mL, and 2.0 µg/mL against *C. glabrata*, *C. lusitaniae*, *C. guilliermondii*, and *C. albicans*, respectively. The MIC_80_ values at 48 h of fluconazole were >16 µg/mL against *C. glabrata* and 1.25 µg/mL for the other strains.

Our results were consistent with previous investigations reporting that the antifungal activity was mainly determined by the presence of a terpenic unit and hydroxyl groups in the aromatic ring [28,29,30], where these induced oxidative stress and compromised the antioxidant defense system in strains of the *Candida* genus [31].

The Quantitative structure–activity relationship (QSAR) models of the compounds synthesized for *C. glabrata*, *C. lusitaniae*, *C. guillermondii*, and *C. albicans* were developed using the multiple linear regression technique with pMIC (-log(MIC)) as a dependent variable and each of the descriptors as an independent variable (see Supplementary Material). The multivariable models for each of the yeasts, *C. glabrata* (Equation (1)), *C. lusitaniae* (Equation (2)), *C. guillermondii* (Equation (3)), and *C. albicans* (Equation (4)), are detailed below:

pMIC = 4.89 (0.21) + 1.78 (0.33)C_5_ + 4.46 (2.14)C_6_ + 12.36 (5.59)C_6_^2^, (1)

N = 8; r = 0.971; r^2^ = 0.943; SD = 0.183; F = 21.99; q^2^ = 0.942.

pMIC = 5.64(0.03) + 2499(461)LUMO^2^ − 0.66 (0.02)C_1_ + 2.56(0.44)C_1_^2^, (2)

N = 8; r = 0.985; r^2^ = 0.971; SD = 0.041; F = 43.97; q^2^ = 0.999.

pMIC = 6.92 (0.30) − 7.72 (1.33)L-H + 45.24 (9.05)C_8’_ − 2.08 (0.33)C_2_^2^, (3)

N = 8; r = 0.995; r^2^ = 0.990; SD = 0.025; F = 134.32; q^2^ = 0.989.

pMIC= 3.30 (1.38) + 10^−5^ *[0.629 (0.29)WI^2^ +59.5 (0.44)MTI − 0.013 (0.006)MTI^2^], (4)

N = 8; r = 0.912; r^2^ = 0.832; SD = 0.127; F = 60.20; q^2^ = 0.831.

Considering all 2D-QSAR equations obtained for the carveoylphenols, the MIC in each of the evaluated *Candida* strains was linked to different descriptors (see Equations (1)–(4)). Moreover, measurements of the partial atomic charges across different carbon skeletons (C_3_, C_5_, C_6_, C_6_^2^, C_1’_, and C_5’_; see Equations (1)–(3)), provided insight into the molecular charge distribution [32] and had been used in QSAR studies [33,34]. In addition, the LUMO ((Lowest Unoccupied Molecular Orbital) descriptor, see Equation (2).) from the physical point of view corresponded to the capacity of a molecule to accept a pair of electrons, analogous to a Lewis acid [35], and had been used as a descriptor to explain the antifungal activity of N-heterocyclic-thioamides, likewise, the L-H descriptor corresponded to the difference between LUMO and HOMO (Highest Occupied Molecular Orbital), which had also been used to explain the antifungal activity [36]. Furthermore, the Wiener index (WI) sums the distances between the molecule vertices [37], while the Molecular Topological Index (MTI) is derived from the molecular connectivity [38] and the Wiener index [39].

In the case of *C. glabrata*, the inhibition of this fungus was mainly influenced by the partial atomic charges in the C_5_ and C_6_ located in the aromatic fragment in positions—para and meta, respectively—for the carbon that was bound to the terpenic portion. Of these atomic charges, C6^2^ was the most important (6.9 times more important than C_5_ and 2.8 times more important than C_6_; see Equation (1). Thus, the modification of the charge in this carbon would allow the development of new *C. glabrata* inhibitor compounds. For example, the most active compounds on this strain corresponded to **5** and **6**, which had a high negative charge density in C_6_, thus increasing the inhibitory activity on *C. glabrata* (Supplementary Material, Table S1). In addition, new derivatives were calculated with substituents in carbon C_6_ based on compound **6**, finding that the addition of fluoride in this position increased activity (pMIC = 7.571, Supplementary Material, Table S2).

In the inhibition caused by cyclodiprenyl phenols derivatives against *C. lusitaniae*, the effect of the LUMO^2^ descriptors and the partial loads in carbon C_1_ and C_12_, corresponding to the carbon in position -*orto* to the carbon bound to the terpenic fragment (see Equation (2)), could be appreciated. Of the three descriptors that can modify the inhibitory activity of *C. lusitaniae*, the LUMO^2^ descriptor was the most important (3786 times more important than C_1_ and 976 times more than C_12_). Thus, the modification of LUMO^2^ would allow the development of new *C. lusitaniae* inhibitors. For example, the compounds with the best inhibitory activity against *C. lusitaniae* corresponded to compounds **7** and **8**, which showed LUMO^2^ values of 1.30·10^−5^ and 6.45·10^−5^, respectively, (pMIC = 6.060 and 6.056, respectively, Supplementary Material, Table S3). Additionally, new derivatives based on compound **7** were calculated, obtaining subtractor substitutes of electrons such as halogens -Cl and -Br linked to C_4_, which can increase the value of LUMO^2^ compared to compound **7**, thus producing an increase in inhibitory activity on *C. lusitaniae* (pMIC = 7.352 and 7.452, respectively, Supplementary Material, Table S4).

On the other hand, the inhibition of *C. guillermondii* was influenced by the difference between LUMO and HOMO (L-H) and the atomic partial charges of C_2_ and C_7’_ carbon squared, which corresponded to the quaternary carbon linked to a double exocyclic bond and the carbon in ortho position to the carbon linked to the terpenic fragment. Considering the three descriptors linked to the inhibition of *C. guillermondii*, the atomic charge in carbon C_7’_ was 5.9 times greater than L-H and 21.8 times higher than C_2_, thus that the modification of the charge on carbon C_7’_ would allow the development of new and better derivatives to inhibit this fungus (Supplementary Material, Table S5). In effect, new derivatives oriented to increase the positive partial load of C_7’_ carbon were calculated, finding that when an electronegative group linked to the mentioned carbon, an increase in the positive charge was observed, increasing the inhibitory activity of *C. guillermondii* (Supplementary Material, Table S6).

In addition, the inhibitory activity of the compounds on *C. albicans* was influenced by the Wiener index, as was the molecular topological index (MTI; see Equation (4)). In contrast to the descriptors that modify the inhibitory activity in the other strains studied, in *C. albicans*, only the topological descriptors influenced, which was related to the shape of the molecule [37,38,39]. Of the three descriptors, the most important corresponded to MTI, since it was 94.6 times greater than WI^2^ and 4577 higher than MTI^2^, thus the modification of MTI would allow the development of new, more active compounds on *C. albicans*. In this context, compound **7** corresponded to the most active, presenting the highest values of WI and MTI (Supplementary Material, Table S7). Based on these results, new compounds were calculated based on **7**, finding that a substitution at C_4_ carbon with -OMOM, -OAc, or -OCF_3_ groups would increase the inhibitory activity of these derivatives against *C. albicans* (Supplementary Material, Table S8).

**Figure 2:**
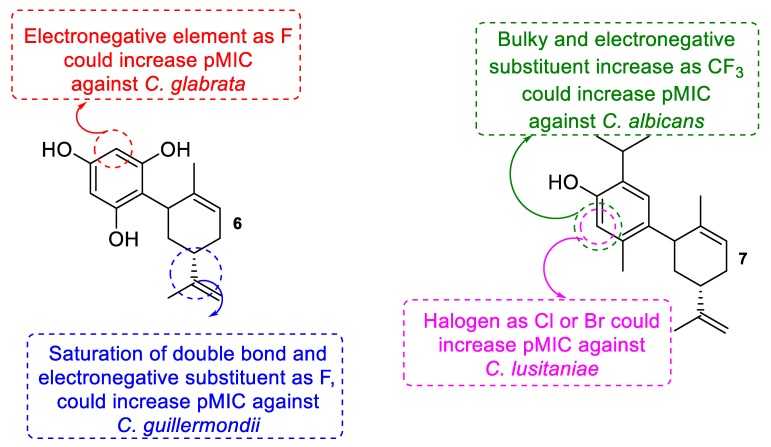
Summary of potential structural modifications to increase the antifungal activity of derivatives **6** and **7** against different strains of *Candida* sp.

Finally, the structural modifications determined to increase the inhibitory activity on the different strains of *Candida* are summarized in Figure 2.

## 3. Materials and Methods 

### 3.1. Chemical

All anhydrous reactions were carried out under nitrogen atmosphere. Solvents were dried by distillation prior to use. Solvent mixtures employed in chromatography were reported as volume to volume ratios. Starting materials were purchased from Aldrich (analytical reagent grades) and used without further purification. Analytical thin-layer chromatography (TLC) was conducted on Merck glass plates coated with silica gel 60 F254 and spots were visualized with UV light and/or an aqueous solution of sulfuric acid (25% *p/p*). Flash column chromatography was performed using Merck silica gel 60 (230–400 mesh the American Society of Testing Materials (ASTM)). Melting points were determined on a Büchi melting point apparatus and were uncorrected. Infrared spectra were obtained on a Thermo Scientific Nicolet 6700 FT-IR spectrometer. HRMS were recorded in a Thermo Finnigan MAT 95 XL mass spectrometers. ^1^H-, ^13^C-, ^13^C DEPT-135, gs 2D HSQC and gs 2D HMBC NMR spectra were recorded on a Bruker Advance 400 MHz. The coupling constants were recorded in Hertz (Hz) and the chemical shifts were reported in parts per million (*δ*, ppm), downfield from tetramethylsilane (TMS) that was used as an internal standard. The % purity of compounds **2**–**9** (**2** (98%), **3** (95%), **4** (92%), **5** (98%), **6** (97%), **7** (96%), **8** (98%), and **9** (96%)) were confirmed by analytical HPLC.

### 3.2. Synthesis

A TiCl_4_ (0.3 mL, 2.73 mmol) was slowly added dropwise to a solution of a phenol derivative (3.3 mmol) and carveol (500 mg, 3.3 mmol) in a suspension of basic aluminum oxide (3 g) in acetonitrile (10 mL), with stirring for 24 h at room temperature and under a N_2_ atmosphere. The reaction was quenched with 10% aqueous solution of sodium bicarbonate (20 mL) and extracted with ethyl acetate (3 × 20 mL). The organic layer was washed with brine, dried, and evaporated to dryness.

*2-[(5R)-5-isopropenyl-2-methylcyclohex-2-en-1-yl]benzene-1,3-diol (**2**)*. Pale orange viscous oil. Yield: 89.0%. Spectroscopic data and physical properties of compound **2** were consistent with those reported in the literature [40].

*2-[(5R)-5-isopropenyl-2-methylcyclohex-2-en-1-yl]benzene-1,4-diol (**3**)*. Pale brown viscous oil, racemic. Yield: 46.8%. IR υ_max_ (KBr) cm^−1^: 3426 (O-H), 2920 (C-H), 1622 (C=C), 1322 (C-H). ^1^H NMR (400.1 MHz, CDCl_3_): 6.66 (d, *J* = 8.4 Hz, 1H, H-3); 6.58 (m, 2H, H-5 and H-6); 5.78 (s, 1H, H-3’); 5.00 (b.s, 1H, OH); 4.71 (s, 1H,OH); 4.65 (m, 2H, H-9’); 3.53 (s, 1H, H-1’); 2.27 (m, 1H, H-5’); 2.06 (m, 2H, H-4’); 1.85 (m, 2H, H-6’); 1.66 (s, 3H, H-8’); 1.63 (s, 3H, H-10’). ^13^C NMR (100.6 MHz, CDCl_3_): 149.4 (C-1); 149.1 (C-4); 147.9 (C-7’); 134.0 (C-2’); 131.0 (C-1); 125.3 (C-3’); 116.8 (C-6); 116.4 (C-3); 113.6 (C-5); 108.7 (C-9); 40.1 (C-1’); 35.3 (C-5’); 34.0 (C-6’); 31.0 (C-4’); 22.5 (C-8’); 20.9 (C-10’). HRMS: M + H ion m/z 245.3368 (calcd for C_16_H_21_O_2_, 245.3367).

*2-[(5R)-5-isopropenyl-2-methylcyclohex-2-en-1-yl]-3,6-dimethylphenol (**4**)*. Yellow viscous oil, racemic. Yield: 38.5%. IR υ_max_ (KBr) cm^−1^: 3339 (O-H), 2935 (C-H), 1620 (C=C), 1322 (C-H). ^1^H NMR (400.1 MHz, CDCl_3_): 7.03 (d, *J* = 7.5 Hz, 1H, H-4); 6.69 (d, *J* = 7.5 Hz, 1H, H-5); 5.76 (s, 1H, H-3’); 4.72 (m, 2H, H-9’); 4.60(s, 1H, OH); 3.45 (b.s, 1H, H-1’); 2.49 (m, 1H, H-5’); 2.35 (s, 3H, H-8); 2.19 (s, 3H, H-7); 2.14 (m, 2H, H-4’); 1.94 (m, 1H, H-6_β_’); 1.84 (s, 3H, H-8’); 1.81 (m, 1H, H-6_α_’); 1.74 (s, 3H, H-10’). ^13^C NMR (100.6 MHz, CDCl_3_): 156.7 (C-1); 149.2 (C-7’); 136.4 (C-2’); 132.4 (C-3); 130.6 (C-5); 126.6 (C-2); 125.0 (C-3’); 121.1 (C-4); 120.7 (C-6); 109.0 (C-9’); 41.0. (C-1’); 36.0 (C-5’); 32.8 (C-6’); 31.1 (C-4’); 21.0 (C-7); 20.9 (C-8’); 20.3 (C-10’); 16.1 (C-8). HRMS: M + H ion m/z 257.3907 (calcd for C_18_H_25_O, 257.3905).

*6-[(5R)-5-isopropenyl-2-methylcyclohex-2-en-1-yl]-5-methylbenzene-1,3-diol (**5**)*. Yellow viscous oil, racemic. Yield: 38.5%. IR υ_max_ (KBr) cm^−1^: 3456 (O-H), 2929 (C-H), 1625 (C=C), 1319 (C-H). ^1^H NMR (400.1 MHz, CDCl_3_): 6.26 (s, 1H, H-4); 6.17 (s, 1H, H-2); 5.91 (b.s, 1H, H-3’); 4.76 (s, 1H, H-9_β_’); 4.72 (s, 1H, OH); 4.70 (s, 1H, H-9_α_’); 3.46 (b.s, 1H, H-1’); 2.40 (m, 1H, H-5’); 2.36 (m, 1H, H-6_β_’); 2.25 (s, 3H, H-7); 2.13 (m, 1H, H-6_α_’); 1.92 (m, 1H, H-4_β_’); 1.80 (m, 1H, H-4_α_’); 1.73 (s, 3H, H-8’); 1.62 (s, 3H, H-10’). ^13^C NMR (100.6 MHz, CDCl_3_): 157.0 (C-1); 154.5 (C-3); 148.1 (C-7’); 138.9 (C-2’); 136.7 (C-5); 126.8 (C-3’); 119.0 (C-1); 110.0 (C-4); 109.2 (C-9); 101.8 (C-2); 38.1 (C-1’); 36.9 (C-5’); 33.8 (C-6’); 30.5 (C-4’); 22.3 (C-8’); 21.2 (C-7); 20.5 (C-10’). HRMS: M + H ion m/z 259.3636 (calcd for C_17_H_23_O_2_, 259.3633).

*2-[(5R)-5-isopropenyl-2-methylcyclohex-2-en-1-yl]benzene-1,3,5-triol (**6**)*. Dark yellow viscous oil, racemic. Yield: 43.1%. IR υ_max_ (KBr) cm^−1^: 3460 (O-H), 2925 (C-H), 1623 (C=C), 1318 (C-H). ^1^H NMR (400.1 MHz, CDCl_3_): 5.90 (s, 2H, H-3 and H-5); 5.01 (s, 1H, OH); 4.92 (s, 1H, OH); 4.71 (m, 2H, H-9’); 3.71 (b.s, 1H, H-1’); 2.33 (m, 2H, H-5 and H-6_β_’); 2.06 (m, 1H, H-6_α_’); 1.85 (m, 2H, H-4’); 1.77 (s, 3H, H-8’); 1.62 (s, 3H, H-10’). ^13^C NMR (100.6 MHz, CDCl_3_): 158.0 (C-6); 155.1 (C-1 and C-3); 148.8 (C-7’); 136.9 (C-2’); 127.4 (C-3’); 109.0 (C-9’); 107.8 (C-1); 96.7 (C-5); 95.4 (C-3); 36.8 (C-1’); 35.8 (C-5’); 34.6 (C-6’); 31.0 (C-4’); 22.5 (C-8’); 20.9 (C-10’). HRMS: M + H ion m/z 261.3363 (calcd for C_16_H_21_O_3_, 261.3361).

*4-[(5R)-5-isopropenyl-2-methylcyclohex-2-en-1-yl]-2-isopropyl-5-methylphenol (**7**)*. Brown viscous oil racemic. Yield: 45.1%. IR υ_max_ (KBr) cm^−1^: 3245 (O-H), 2925 (C-H), 1621 (C=C), 1215 (C-H). ^1^H NMR (400.1 MHz, CDCl_3_): 6.91 (s, 1H, H-5); 6.57 (s, 1H, H-2); 5.73 (b.s, 1H, H-3’); 4.71 (s, 1H, H-9_β_’); 4.60 (s, 1H, H-9_α_’); 4.52 (b.s, 1H, OH); 3.45 (s, 1H, H-1’); 3.14 (q, *J* = 6.9 Hz, 1H, H-7); 2.32 (m, 1H, H-5’); 2.27 (s, 3H, H-10); 2.17 (m, 2H, H-4_β_’ and H-6_β_’); 1.93 (m, 1H, H-4_α_’); 1.84 (m, 1H, H-6_α_’); 1.75 (s, 3H, H-10’); 1.64 (s, 3H, H-10’); 1.22 (s, 3H, H-8); 1.21 (s, 3H, H-10). ^13^C NMR (100.6 MHz, CDCl_3_): 150.4 (C-7’); 150.0 (C-1); 134.7 (C-2’); 134.4 (C-4); 130.8 (C-3 and C-6); 126.2 (C-5); 123.9 (C-3’); 117.4 (C-2); 108.4 (C-9’); 41.2 (C-1’); 35.0 (C-5’); 34.2 (C-6’); 31.1 (C-4’); 26.8 (C-7); 22.8 (C-8 and C-9); 22.6 (C-8’); 20.8 (C-10’); 18.7 (C-10). HRMS: M + H ion m/z 285.4440 (calcd. for C_20_H_29_O, 285.4436).

*4-[(5R)-5-isopropenyl-2-methylcyclohex-2-en-1-yl]-2,6-dimethoxyphenol (**8**)*. Dark yellow viscous oil racemic. Yield: 48.8%. IR υ_max_ (KBr) cm^−1^: 3460 (O-H), 2926 (C-H), 1619 (C=C), 1321 (C-O), 1215 (C-H). ^1^H NMR (400.1 MHz, CDCl_3_): 6.42 (s, 2H, H-3 and H-5); 5.71 (b.s, 1H, H-3’); 4.71 (s, 1H, OH); 4.64 (m, 2H, H-9’); 3.88 (s, 6H, 2xOCH_3_); 3.27 (b.s, 1H, H-1’); 2.19 (m, 2H, H-5 and H-4_β_’); 1.90 (m, 2H, H-4_α_’and H-6_β_’); 1.76 (m, 1H, H-6_α_’); 1.64 (s, 3H, H-8’); 1.61 (s, 3H, H-10’). ^13^C NMR (100.6 MHz, CDCl_3_): 149.7 (C-7’); 146.8 (C-2 and C-6); 136.1 (C-4); 134.0 (C-2’); 132.9 (C-1); 124.1 (C-3); 108.5 (C-9’); 105.2 (C-3 and C-5); 56.3 (2xOCH_3_); 45.7 (C-1’); 36.7 (C-6’); 35.1 (C-5’); 31.0 (C-4’); 22.6 (C-8’); 20.9 (C-10’). HRMS: M + H ion m/z 289.3895 (calcd. for C_18_H_25_O_3_, 289.3893).

*3-[(5R)-5-isopropenyl-2-methylcyclohex-2-en-1-yl]- 2,6-dimethoxyphenol (**9**)*. Dark brown viscous oil racemic. Yield: 20.0%. IR υ_max_ (KBr) cm^−1^: 3460 (O-H), 2925 (C-H), 1623 (C=C), 1325 (C-O), 1219 (C-H). ^1^H NMR (400.1 MHz, CDCl_3_): 6.58 (s, 2H, H-4 and H-5); 5.71 (b.s, 1H, H-3’); 4.69 (s, 1H, OH); 4.63 (m, 2H, H-9’); 3.92 (s, 3H, OCH_3_); 3.88 (s, 3H, OCH_3_); 3.67 (b.s, 1H, H-1’); 2.31 (m, 1H, H-5’); 2.19 (m, 2H, H-4_β_’ and H-6_β_’); 1.94 (m, 1H, H-4_α_’); 1.86 (m, 1H, H-6_α_’); 1.66 (s, 3H, H-8’); 1.61 (s, 3H, H-10’). ^13^C NMR (100.6 MHz, CDCl_3_): 150.0 (C-2 and C-6); 146.1 (C-7’); 138.5 (C-1); 134.1 (C-2’); 131.4 (C-4); 124.1 (C-3’); 119.0 (C-3); 108.5 (C-9’); 105.5 (C-5); 60.7 (OCH_3_); 56.1 (OCH_3_); 41.9 (C-1’); 38.5 (C-5’); 34.9 (C-6’); 31.1 (C-4’); 22.5 (C-8’); 20.9 (C-10’). HRMS: M + H ion m/z 289.3894 (calcd. for C_18_H_25_O_3_, 289.3893).

### 3.3. Microorganims

The fungal strains used in this work belong to the collection of the Laboratory of Clinical Microbiology, Faculty of Medicine, University of Valparaíso. The collection includes *Candida glabrata* 10912, *C. lusitaniae* 2305, *C. guilliermondii* 2204 and *C. albicans* 10935. Strains were grown by the procedure previously described in reference [41].

### 3.4. Antifungal Assays

The antifungal assay was performed in accordance with the guidelines of the NCCLS [28]. Briefly, yeast strains were prepared in sterile water and were diluted in RPMI 1640 medium (except in the sterility control). All compounds were dissolved in dimethylsulfoxide (DMSO) at final concentrations of 16 to 0.03 µg/mL. Fluconazole and Itraconazole were used as positive controls. The plates were incubated at 37 °C for 24–48 h and the absorbance measured at 540 nm. The results were obtained as the Minimum inhibitory concentration (MIC_80_) values. All MICs readings were accomplished 3 times for each compound employed.

### 3.5. Computational Details

Structures of all the compounds were geometry optimized with Gaussian 09 program [42] using Density Functional Theory (DFT) with B3LYP-6-31G (d,p) basis set. The geometries of all the compounds and the reactivity descriptors used were calculated by the equations previously described in reference [43]. In addition, topological and steric descriptors used [43] were obtained using the Chem-Draw software.

#### Modeling

A quantitative structure–activity relationship (QSAR) study was carried out using multiple linear regressions as previous reported by our research group [44,45]. Briefly, the dependent variable (pMIC; -log10(MIC)) in molar unit was correlated with all the descriptors previously calculated (independent variable) in lineal and square form (WI, WI^2^, SC, SC^2^, ST, ST^2^, R, R^2^, MTI, MTI^2^, D, D^2^, BI, BI^2^, MW, MW^2^, VM, VM^2^, MS, MS^2^, CLogP, CLogP^2^, MR, MR^2^, DM, DM^2^, HOMO, HOMO^2^, LUMO, LUMO^2^, ΔLH, ΔLH^2^, µ, µ^2^, η, η^2^, S, S^2^, ω, and ω^2^) using Statistica 7.0 software. To avoid a random correlation, all models were evaluated with cross-validation using the methodology previously described in reference [43].

## 4. Conclusions

The results suggest that studied carveoylphenols have potential as new antifungal agents against *C. glabrata*, *C. lusitaniae*, *C.* *guilliermondii*, and *C. albicans*. A quantitative structure–activity relationship (QSAR) analysis of the whole series, supported by electronic studies, suggested that **5**, **6**, and **7** compounds have structural features necessary for the design of new compounds with enhanced antifungal activity. Previous studies have shown terpenphenols possess considerable anti-adhesion, anti-biofilm effects, and inhibitory activity on morphogenesis and exoenzyme production of *Candida* species. However, hitherto no clear mechanism of action of these compounds on *Candida* cells and virulence factors has been described compared to the existing antifungal agents. Therefore, these promising results strongly encourage further studies with the purpose of producing new molecules with antimycotic activities in the near future, particularly in the health field.

## Figures and Tables

**Table 1:** Antifungal activities determined in a liquid medium and expressed by ^a^MIC_80_ in μg/mL at 24 h.

Compounds	Microorganism			
	*C. glabrata*	*C. lusitaniae*	*C. guilliermondii*	*C. albicans*
**1**	>16	>16	>16	>16
**2**	>16	0.50	0.5	1.0
**3**	>16	0.125	0.5	0.25
**4**	>16	0.125	1.0	1.0
**5**	2.0	0.50	1.0	0.5
**6**	0.5	0.25	0.5	0.5
**7**	>16	0.25	1.0	0.5
**8**	>16	1.0	1.25	1.25
**9**	>16	0.25	1.25	4.0
Itraconazole	16	2.0	4.0	1.0
Fluconazole	>16	1.0	1.0	1.0
DMSO	i	i	i	i

i, inactive; ^a^MIC_80_ corresponds to the minimum inhibitory concentration of the compound that inhibits 80% of the fungal growth.

**Table 2:** Antifungal activities determined in a liquid medium and expressed by ^a^MIC_80_ in μg/mL at 48 h.

Compounds	Microorganism			
	*C. glabrata*	*C. lusitaniae*	*C. guilliermondii*	*C. albicans*
**1**	>16	>16	>16	>16
**2**	>16	0.50	1.0	2.0
**3**	>16	0.50	0.5	0.5
**4**	>16	0.25	1.25	1.0
**5**	4.0	0.50	1.25	1.0
**6**	1.0	0.50	1.0	1.0
**7**	>16	0.50	1.0	2.0
**8**	>16	1.0	1.50	2.0
**9**	>16	0.50	2.0	8.0
Itraconazole	>16	4.0	8.0	2.0
Fluconazole	>16	1.25	1.25	1.25
DMSO	i	i	i	i

i, inactive; ^a^MIC_80_ corresponds to the minimum inhibitory concentration of the compound that inhibits 80% of the fungal growth.

## Supplementary Materials

The following are available online at https://www.mdpi.com/2079-6382/8/4/185/s1. Figure S1. Structure of phenols, Spectra S1. IR, ^1^H, ^13^C NMR, and HRMS of compounds **3**–**9**, Table S1. Descriptors linked to the inhibition of *C. glabrata* obtained by multivariate analysis, Table S2. Proposed derivatives from compound 6 to improve the inhibitory activity of *C. glabrata*, Table S3. Descriptors linked to the inhibition of *C. lusitaniae* obtained by QSAR analysis, Table S4. Proposed derivatives from compound **7** to improve the inhibitory activity of *C. lusitaniae*, Table S5. Descriptors linked to the inhibition of *C. guillermondii* obtained by multivariate analysis, Table S6. Proposed derivatives from compound **6** to improve the inhibitory activity of *C. guillermondii*, Table S7. Descriptors linked to the inhibition of *C. albicans* obtained by QSAR analysis, Table S8. Proposed derivatives from compound **7** to improve the inhibitory activity of *C. albicans*.
