# Supplementary material for: Carveoylphenols and Their Antifungal Potential against Pathogenic Yeasts
Source: Antibiotics (Basel). 2019 Oct 15;8(4):185. doi: 10.3390/antibiotics8040185 (PMC6963845; doi:10.3390/antibiotics8040185)
Supplement: Supplementary file 1 [file antibiotics-08-00185-s001.pdf]

SUPPORTING INFORMATION

Carveoylphenols and Their Antifungal Potential Against Pathogenic Yeasts

Iván Montenegro [1], Marco Mellado [2], Alessandra Russo [3], Bastian Said [4], Ximena Besoain [5], Patricio Godoy [6], Enrique Werner [7], Nelson Caro [8] and Alejandro Madrid [9,*]

[1] Escuela de Obstetricia y Puericultura, Facultad de medicina, Universidad de Valparaíso, Angamos 655, Reñaca, Viña del Mar 2520000, Chile; ivan.montenegro@uv.cl
[2] Instituto de Química, Facultad de Ciencias, Pontificia Universidad Católica de Valparaíso, Av. Universidad #330, Curauma, Valparaíso2340000, Chile; marco.mellado@pucv.cl
[3] Department of Drug Sciences, University of Catania, Via S. Sofia 64, 95125 Catania, Italy; alrusso@unict.it
[4] Departamento de Química, Universidad Técnica Federico Santa María, Av. Santa María 6400, Vitacura 7630000, Santiago, Chile; bastian.said@usm.cl
[5] Escuela de Agronomía Pontificia Universidad Católica de Valparaíso, Quillota, SanFrancisco s/n La Palma, Quillota 2260000, Chile; ximena.besoain@pucv.cl
[6] Instituto de Microbiología Clínica, Facultad de Medicina, Universidad Austral de Chile, Los Laureles s/n, Isla Teja, Valdivia 5090000, Chile; patricio.godoy@uach.cl
[7] Departamento De Ciencias Básicas, Campus Fernando May, Universidad del Bío-Bío, Avda. Andrés Bello 720, casilla 447, Chillán 3780000, Chile; ewerner@ubiobio.cl
[8] Centro de Investigación Australbiotech, Universidad Santo Tomás, Avda. Ejército 146, Santiago 8320000, Chile; ncaro@australbiotech.cl
[9] Laboratorio de Productos Naturales y Síntesis Orgánica (LPNSO), Departamento de Química, Facultad de Ciencias Naturales y Exactas, Universidad de Playa Ancha, Avda. Leopoldo Carvallo 270, Playa Ancha, Valparaíso, Chile; Alejandro.madrid@upla.cl
* Correspondence: alejandro.madrid@upla.cl; Tel.: +56-032-250-0526

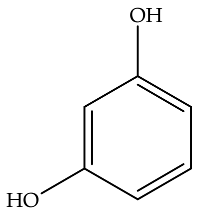

A

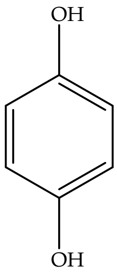

B

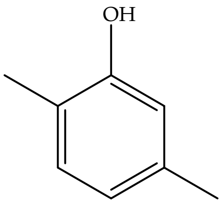

C

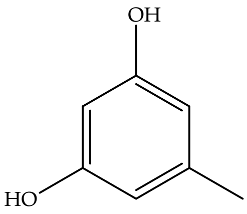

D

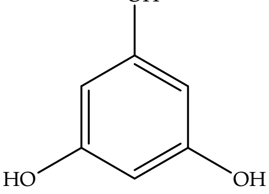

E

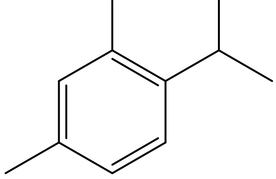

F

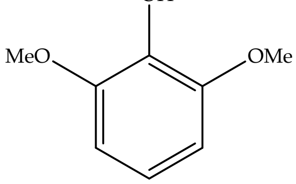

G

Figure S1. Structure of phenols.

SpectraS1: IR, ^{1}H, ^{13}C NMR, and MS of compounds 3–9

IR spectrum of compound 3

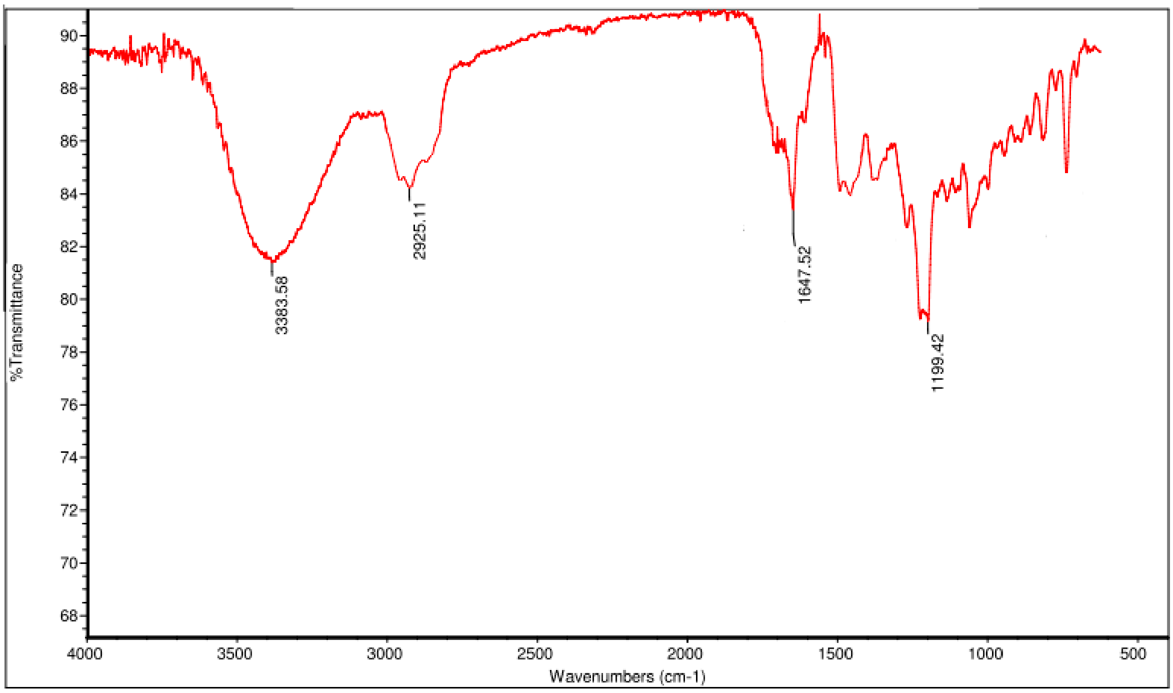

^{1}H NMR (400 MHz, $CDCl_3$) spectrum of compound

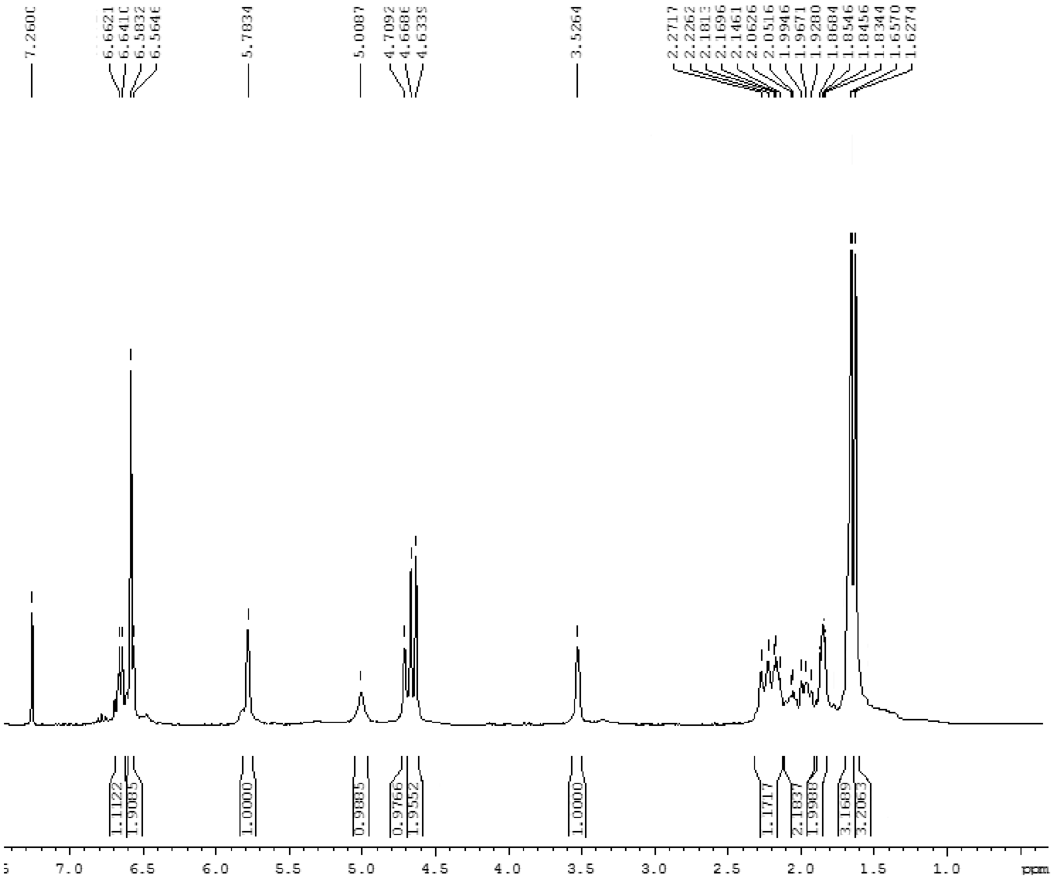

^{13}C NMR (100 MHz, $CDCl_3$) spectrum of compound 3

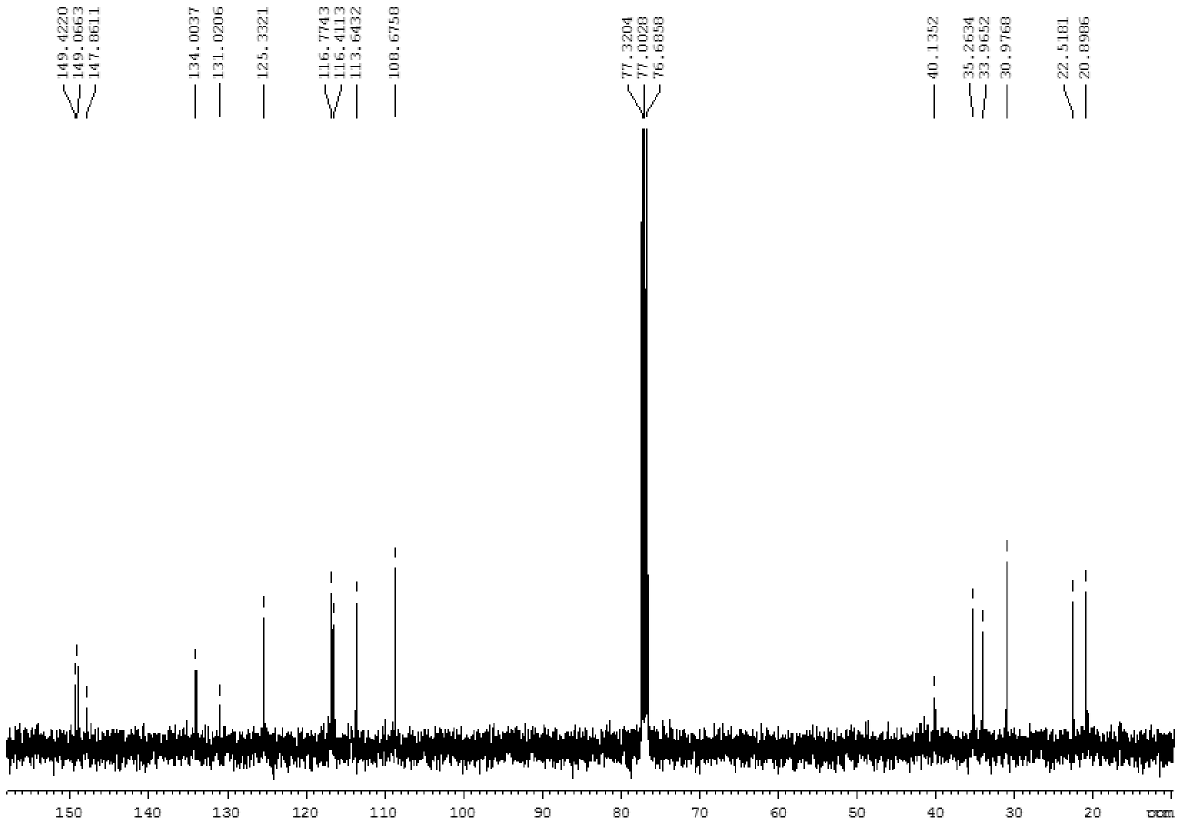

Mass of compound 3

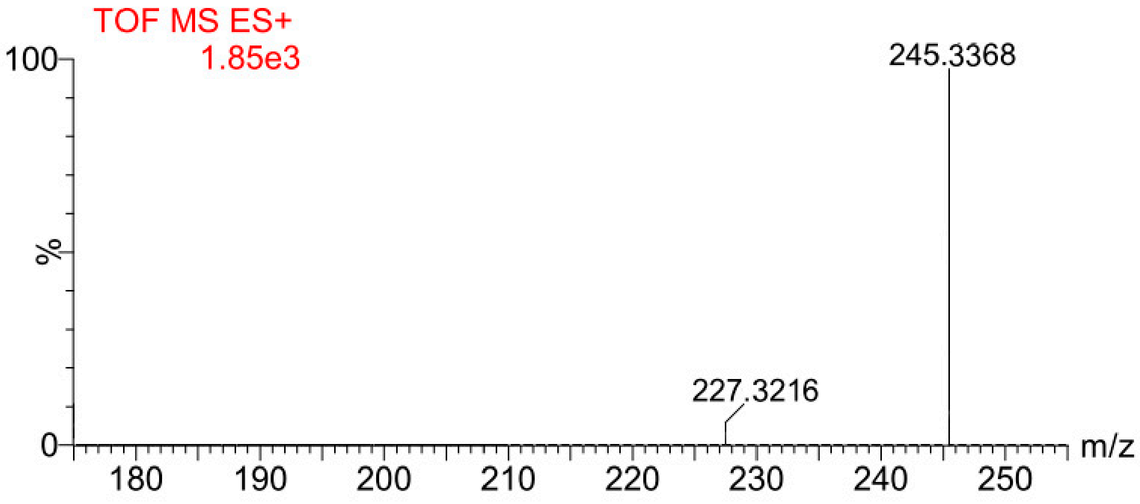

IR spectrum of compound 4

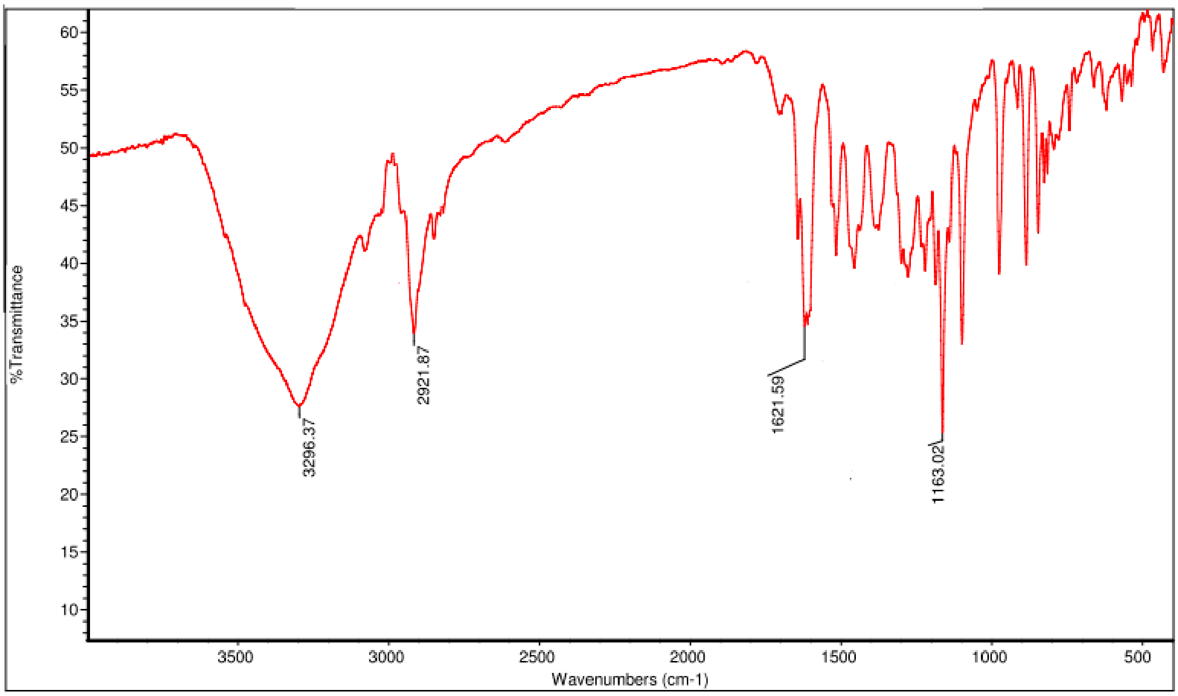

^{1}H NMR (400 MHz, $CDCl_3$) spectrum of compound 4

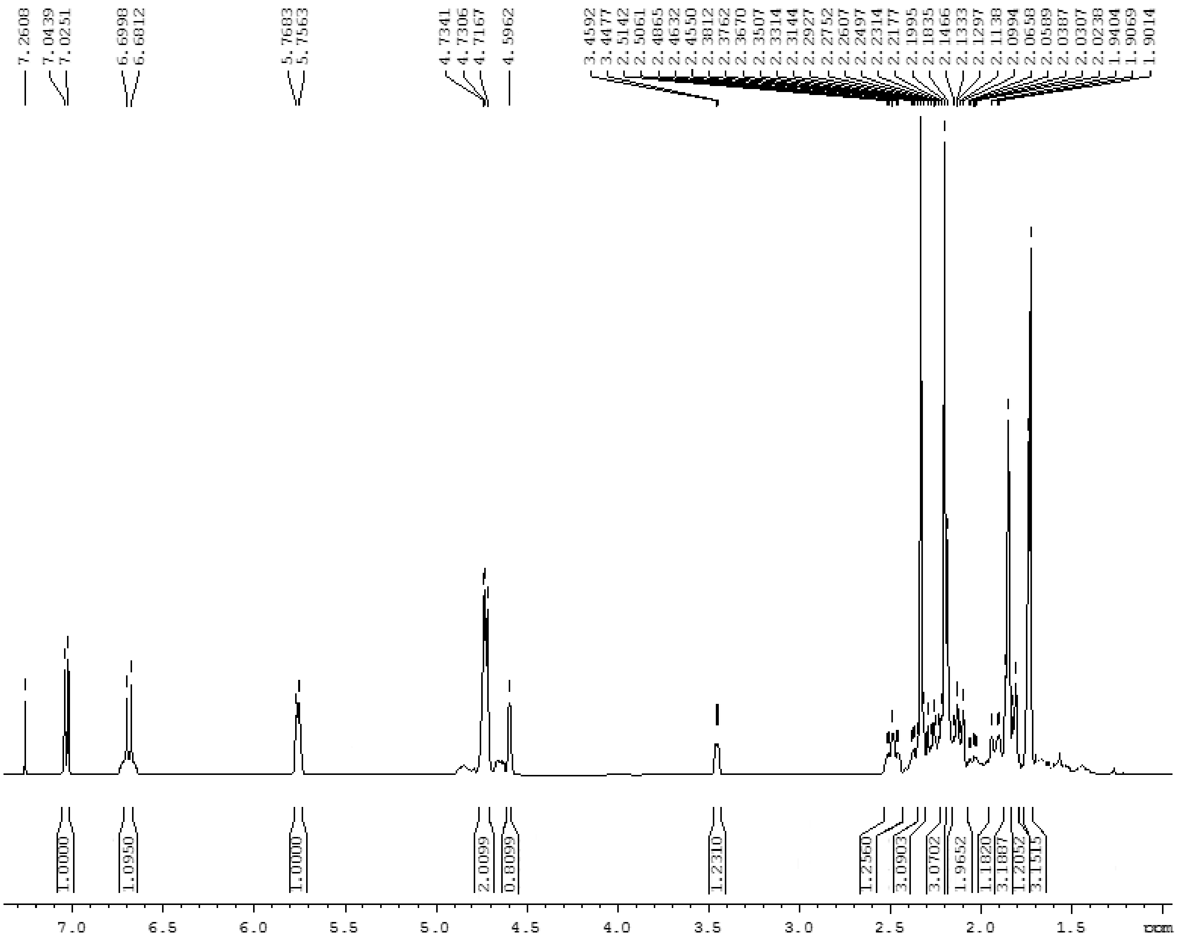

^{13}C NMR (100 MHz, CDCl$_3$) spectrum of compound 4

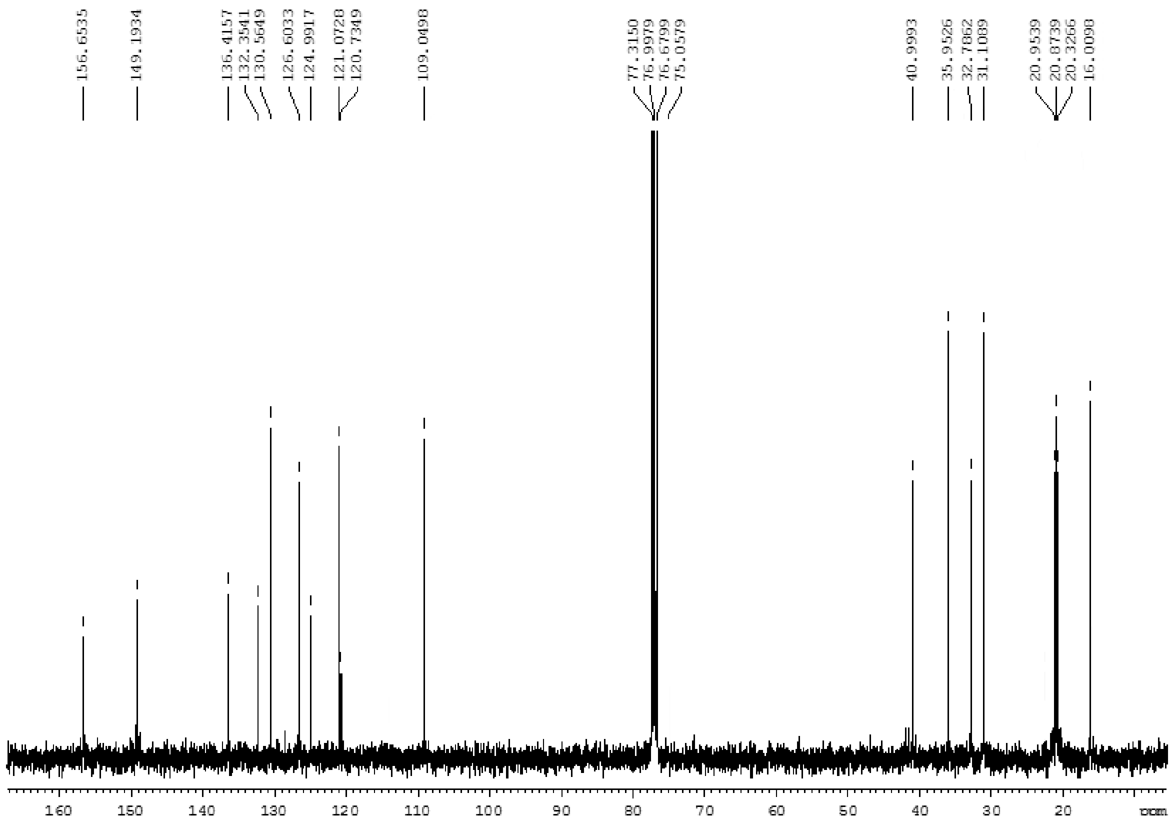

Mass of compound 4

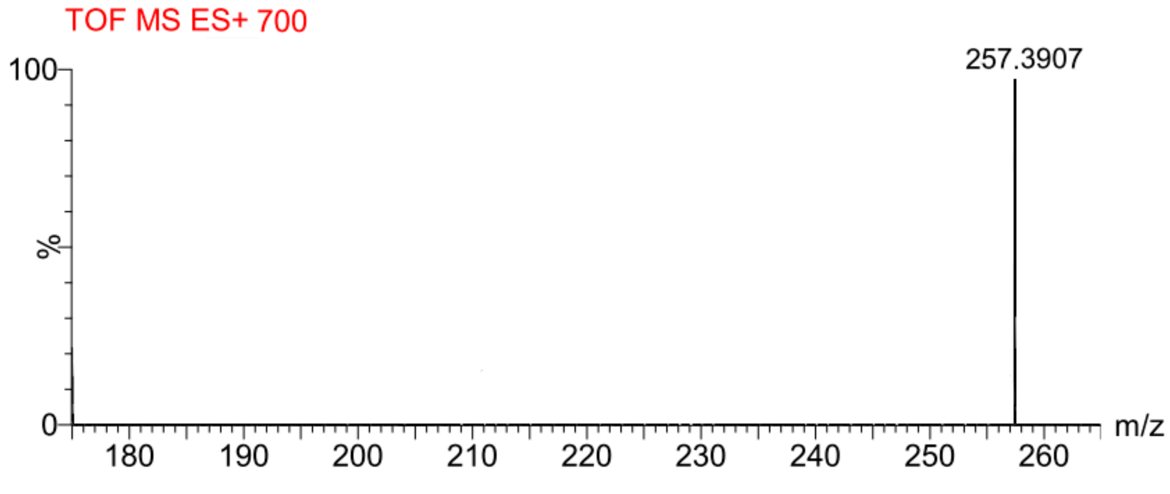

IR spectrum of compound 5

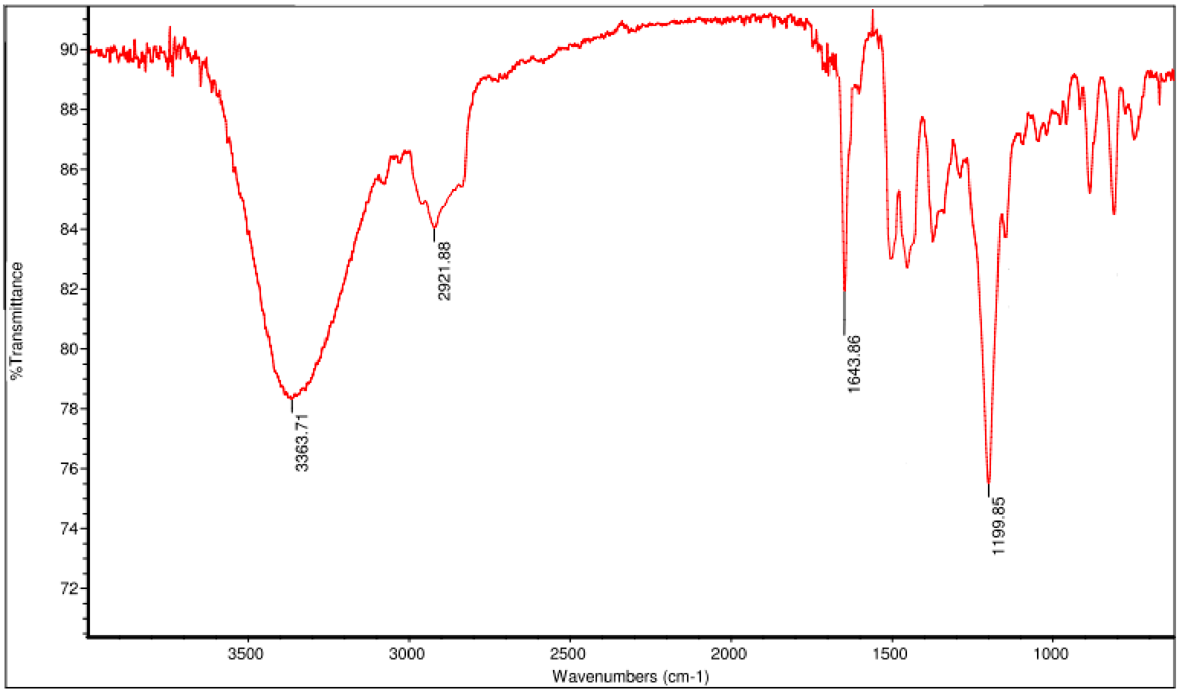

^{1}H NMR (400 MHz, $CDCl_3$) spectrum of compound 5

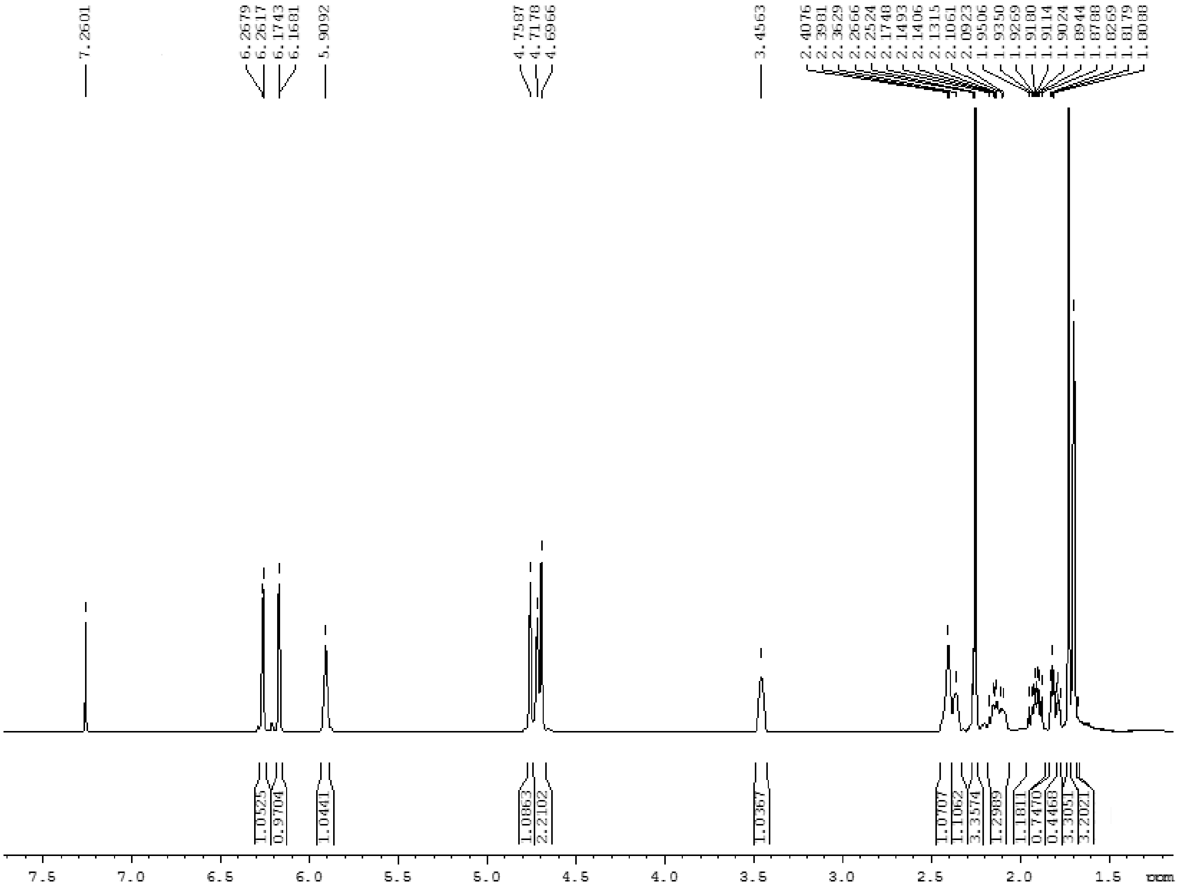

^{13}C NMR (100 MHz, $CDCl_3$) spectrum of compound 5

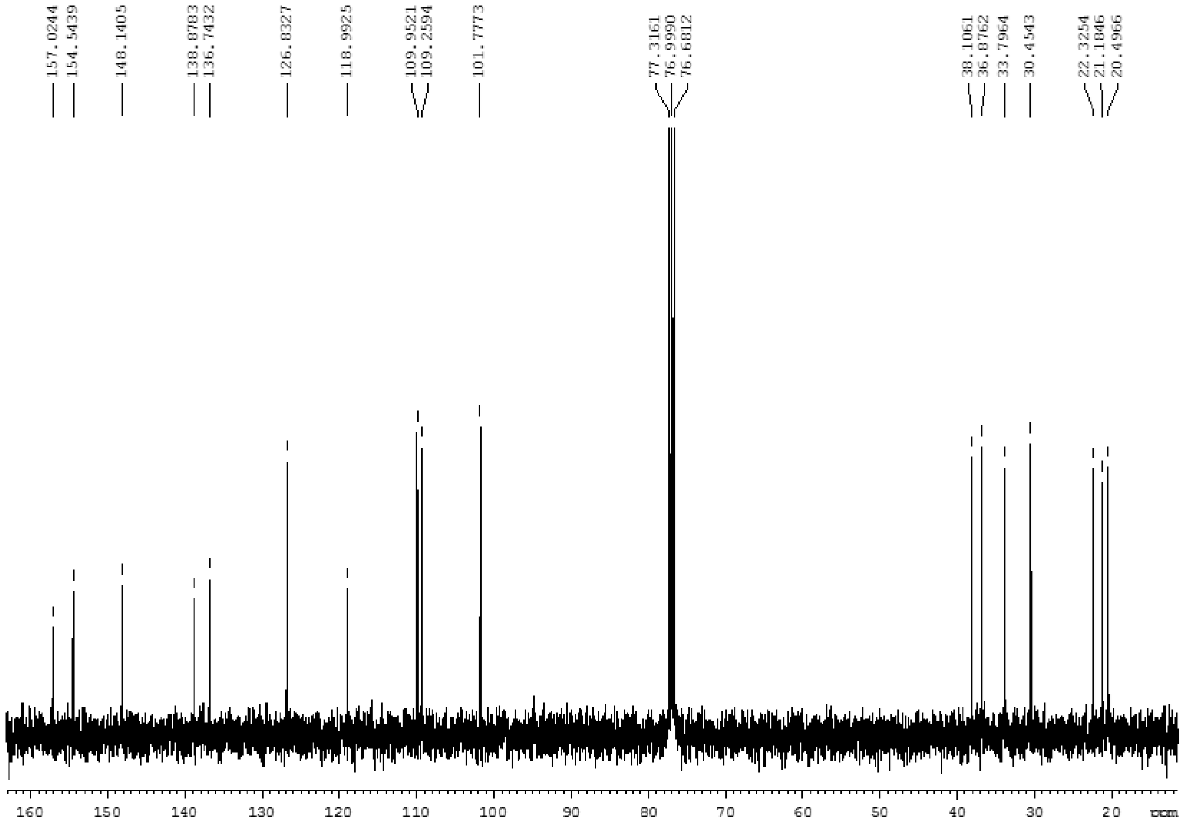

Mass of compound 5

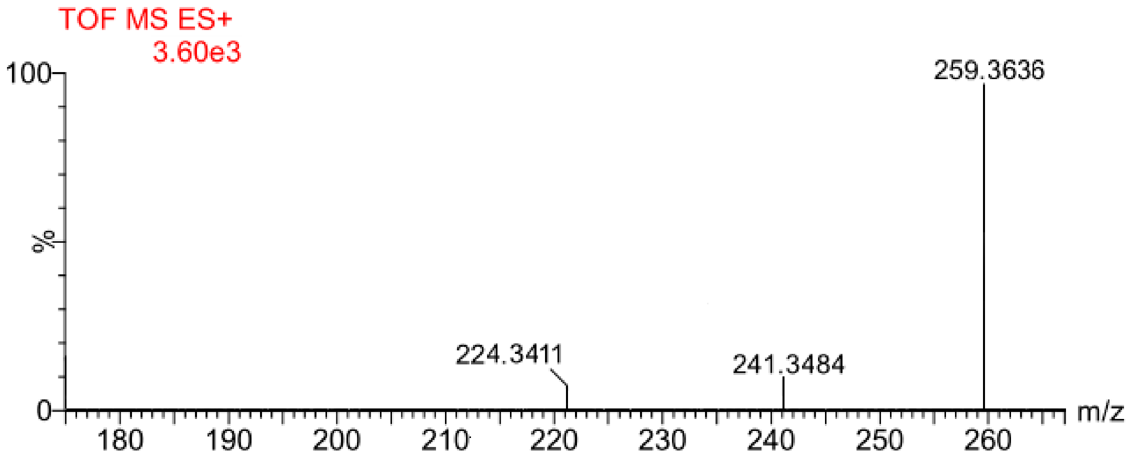

IR spectrum of compound 6

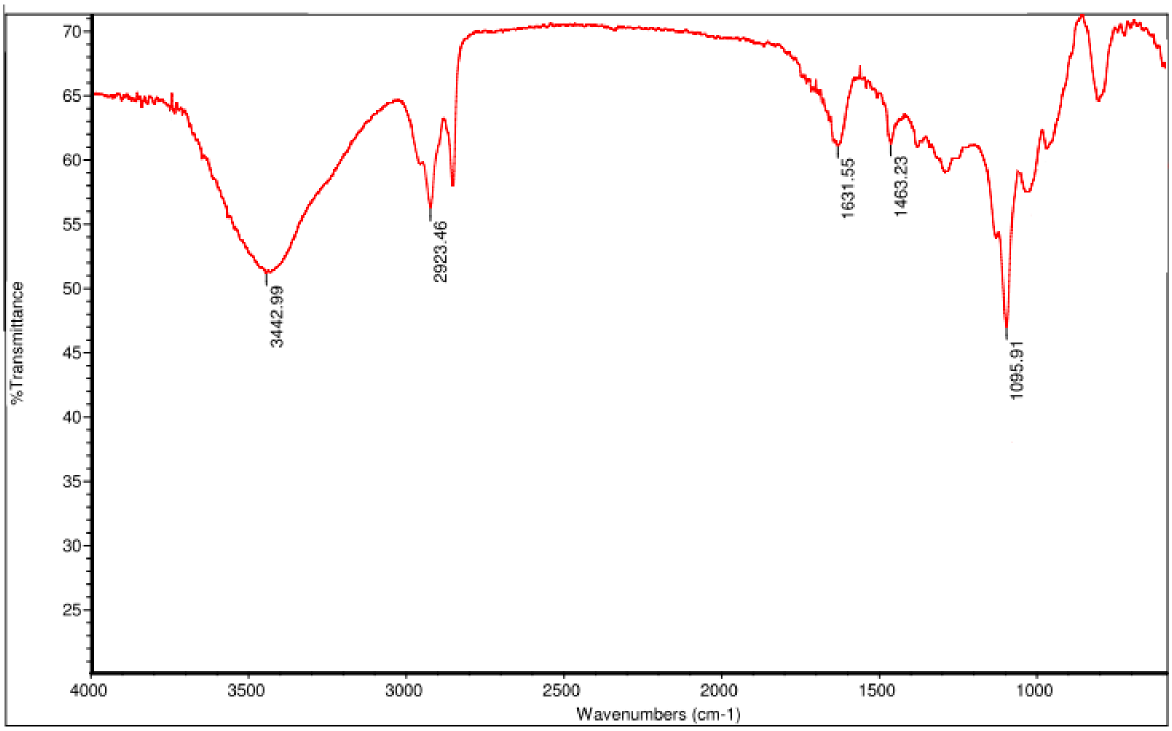

^{1}H NMR (400 MHz, $CDCl_3$) spectrum of compound 6

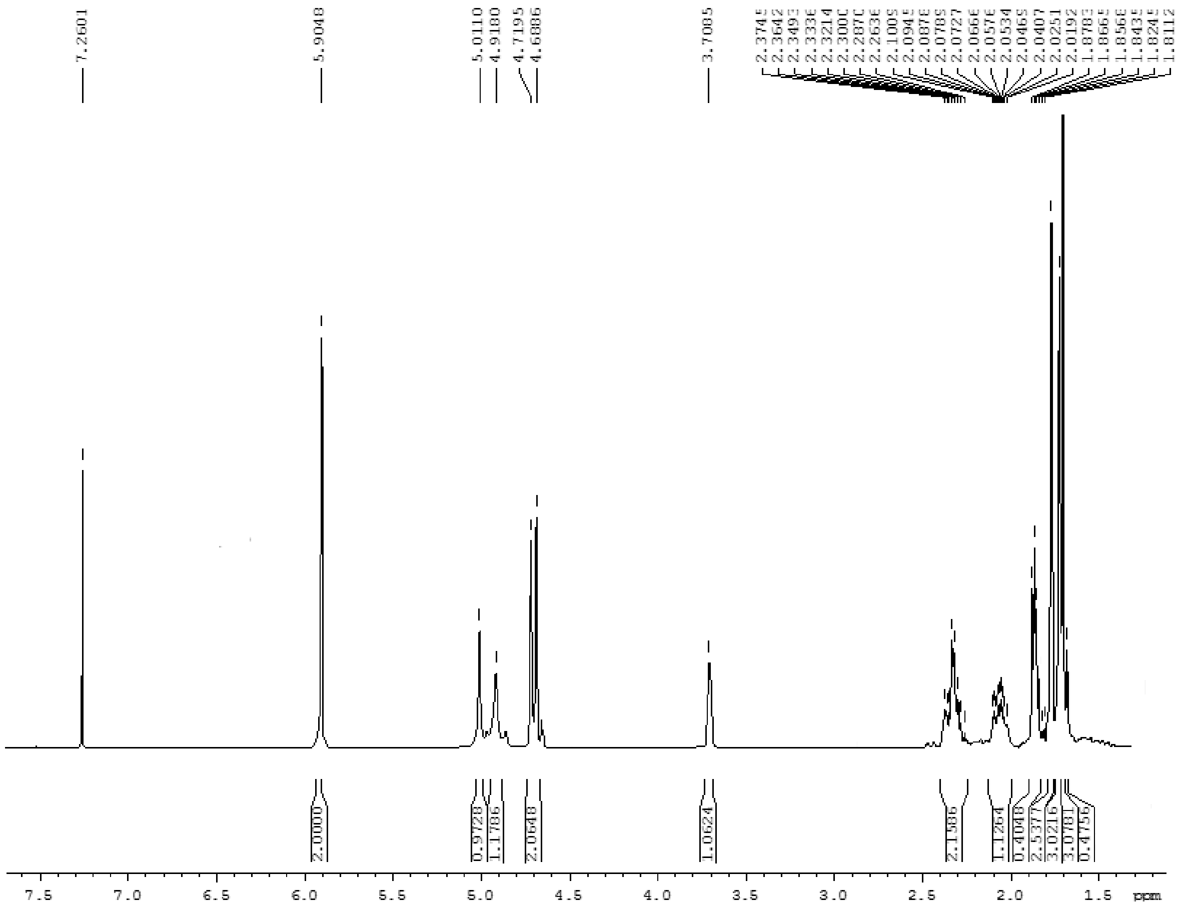

^{13}C NMR (100 MHz, $CDCl_3$) spectrum of compound 6

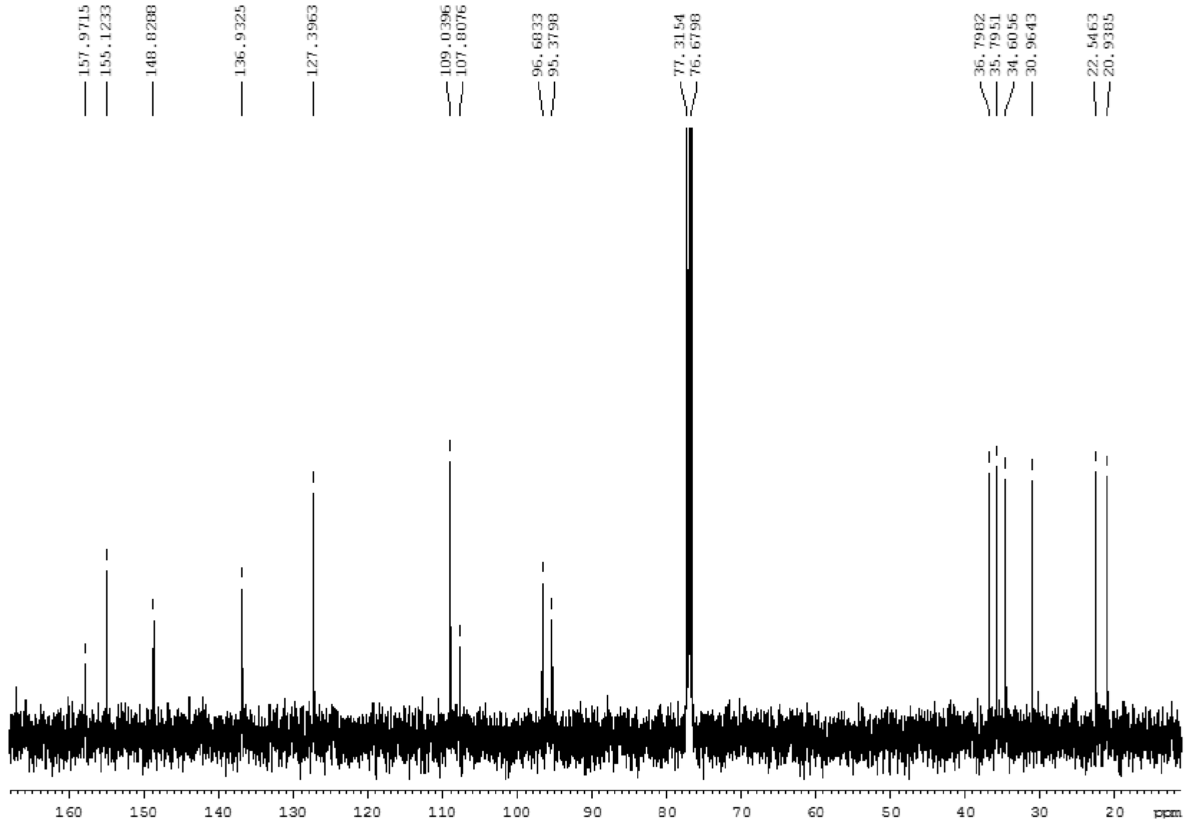

Mass of compound 6

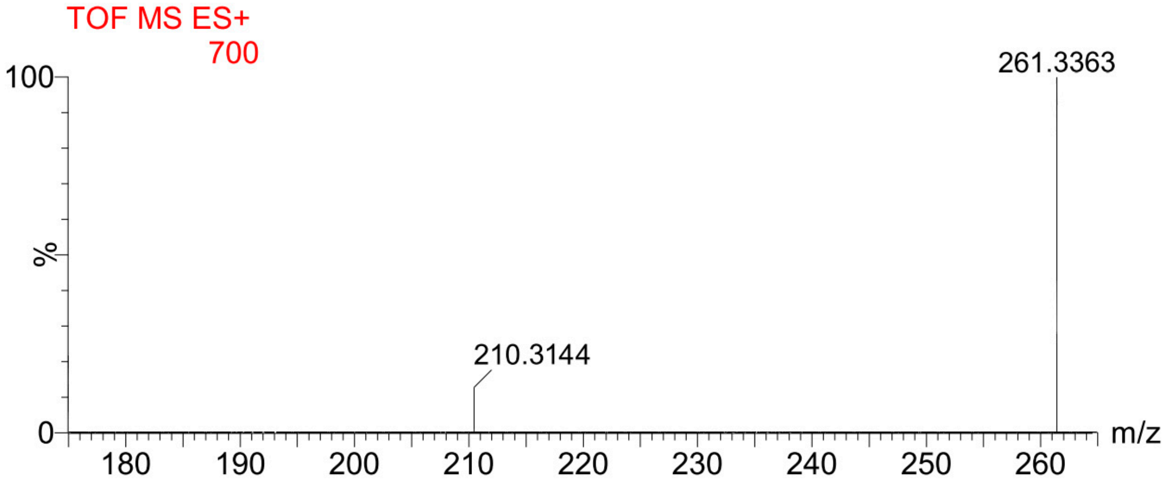

IR spectrum of compound 7

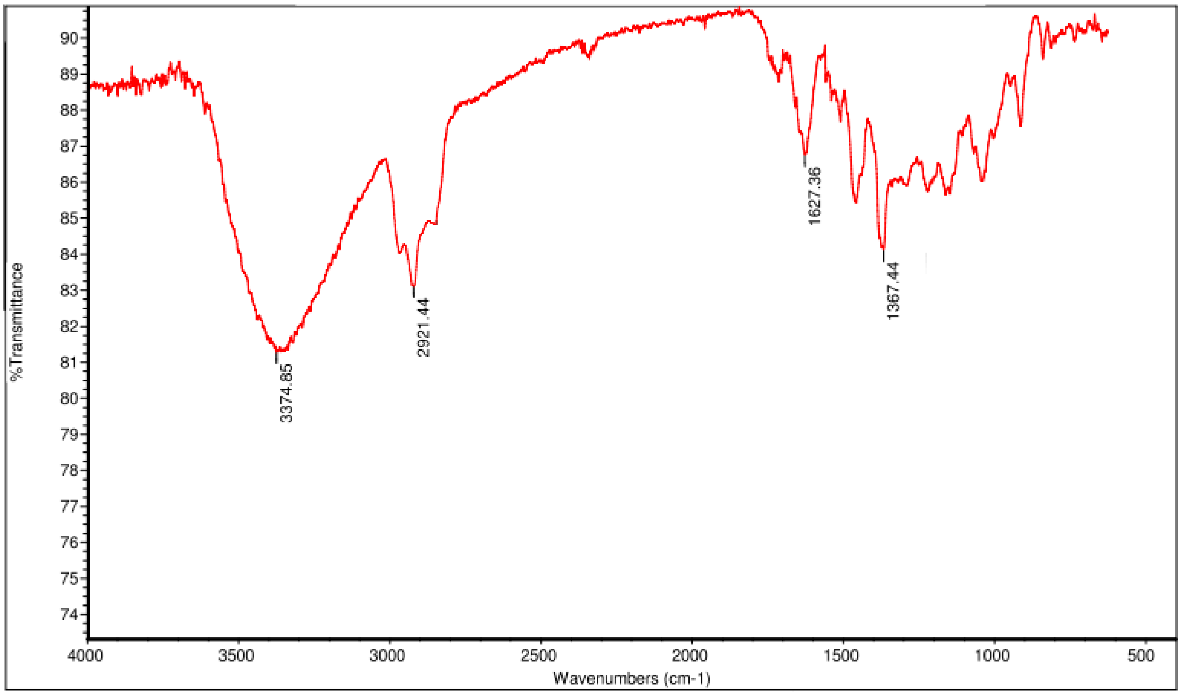

^{1}H NMR (400 MHz, CDCl3) spectrum of compound 7

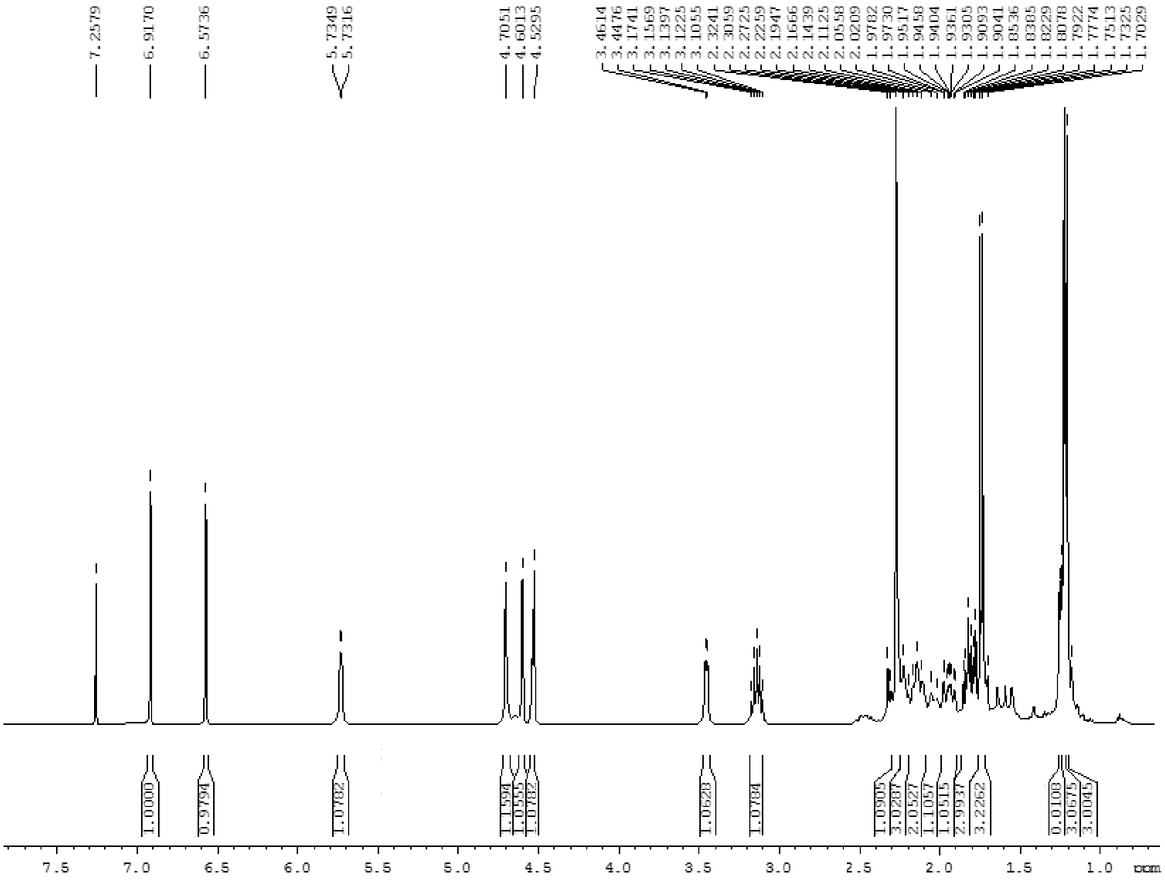

^{13}C NMR (100 MHz, CDCl$_3$) spectrum of compound 7

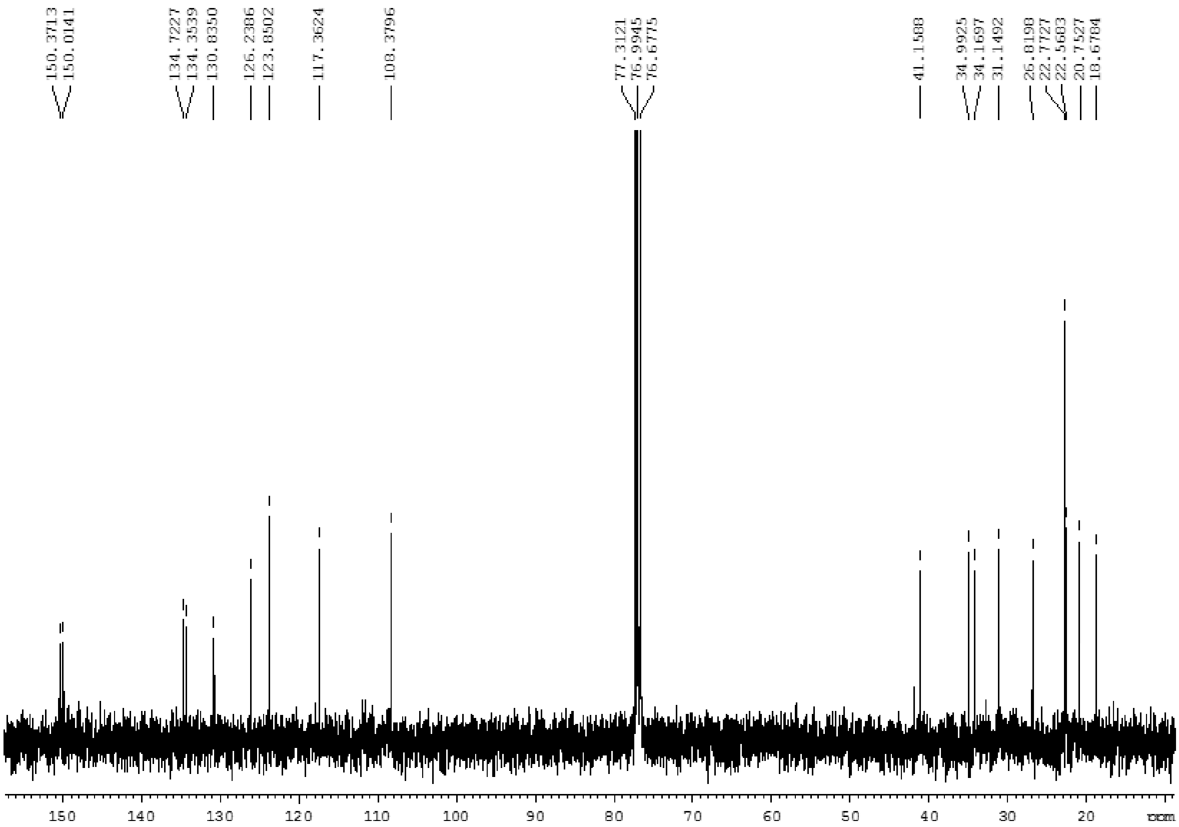

Mass of compound 7

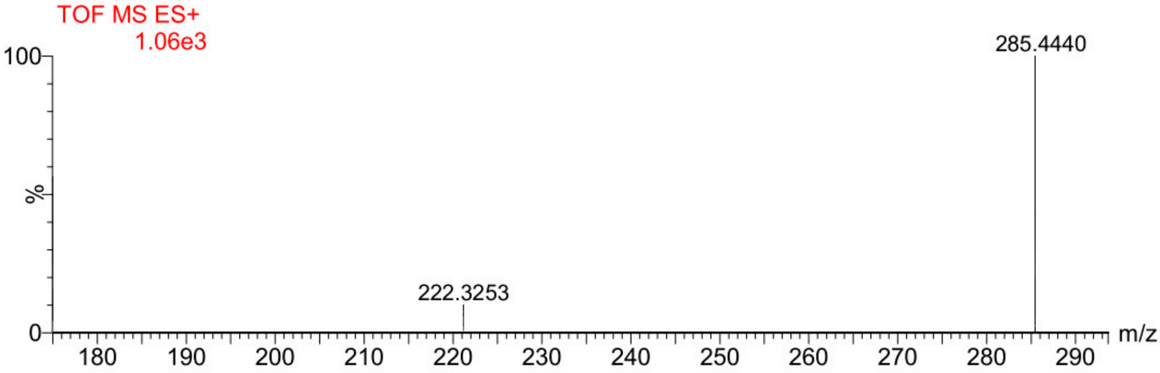

IR spectrum of compound 8

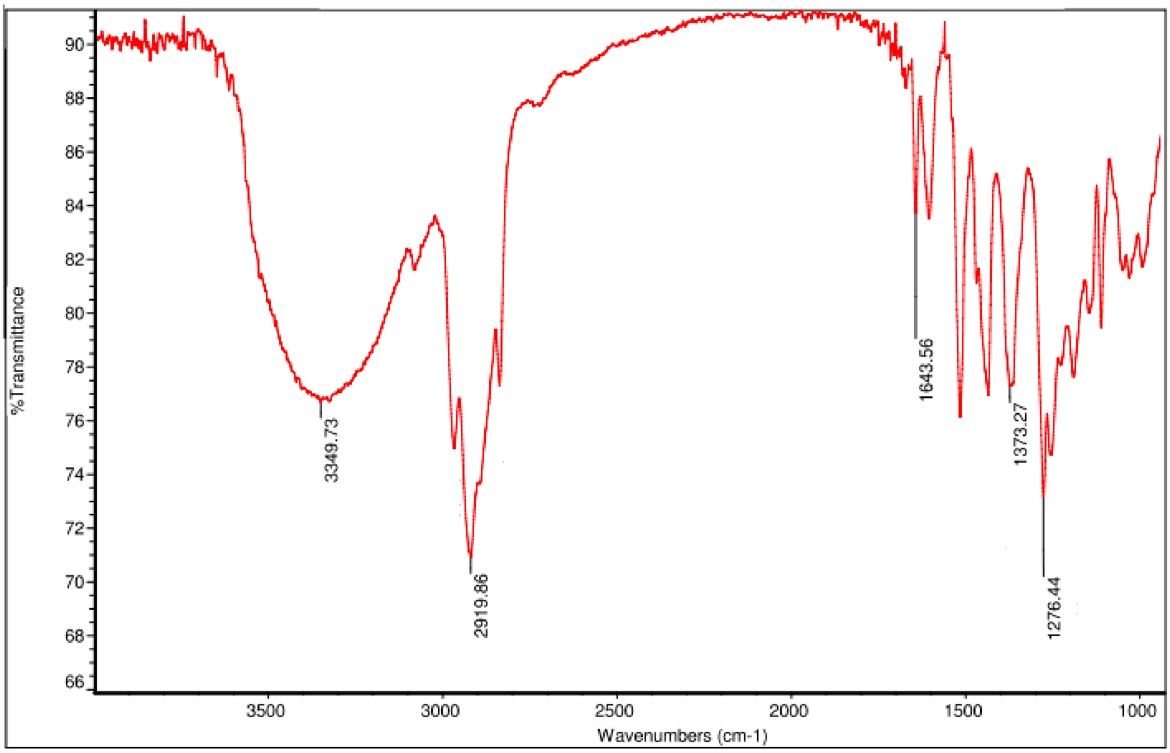

^{1}H NMR (400 MHz, CDCl3) spectrum of compound 8

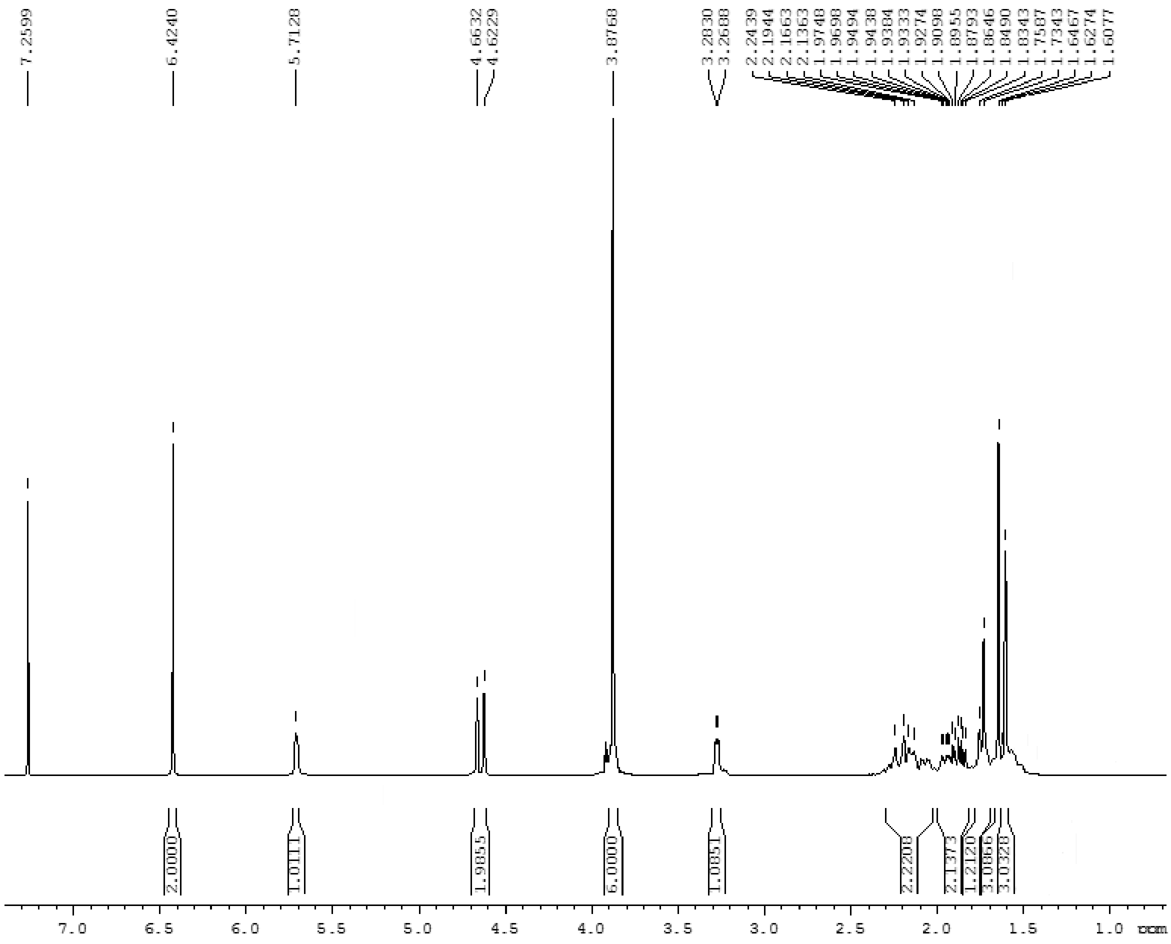

^{13}C NMR (100 MHz, CDCl3) spectrum of compound 8

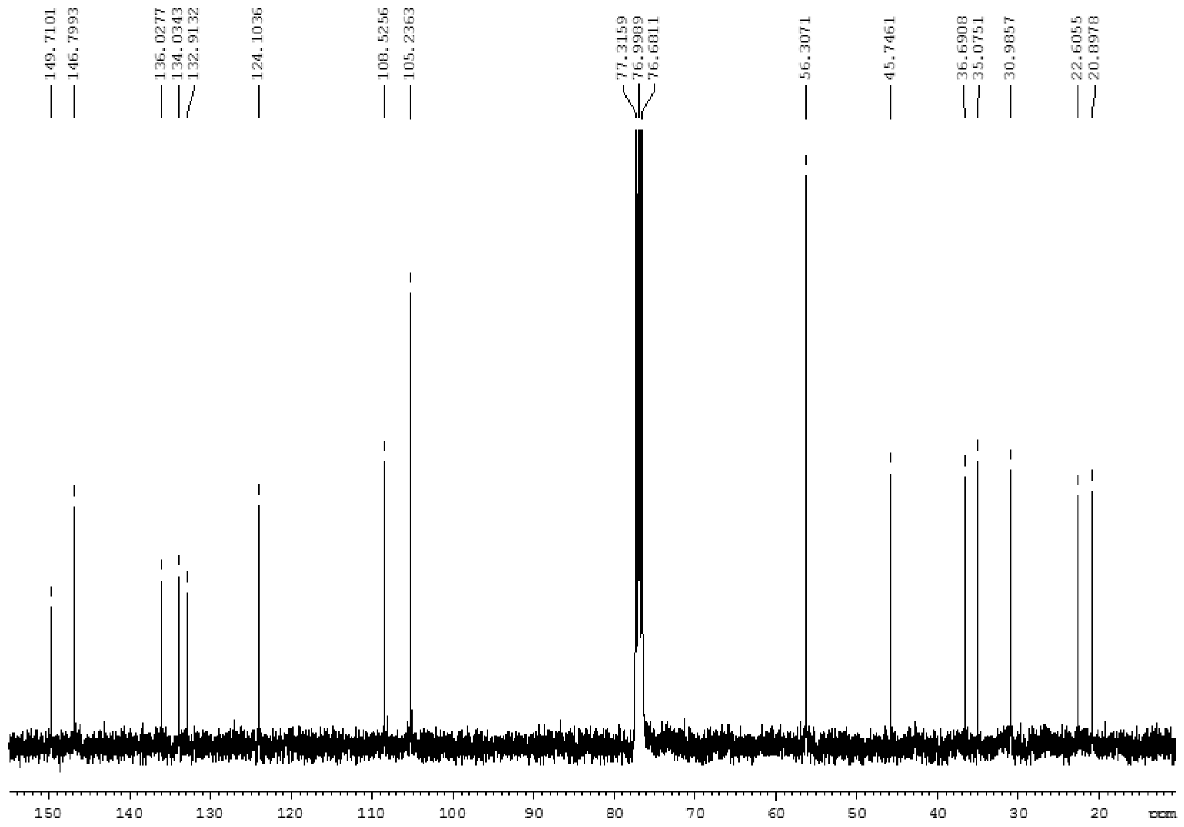

Mass of compound 8

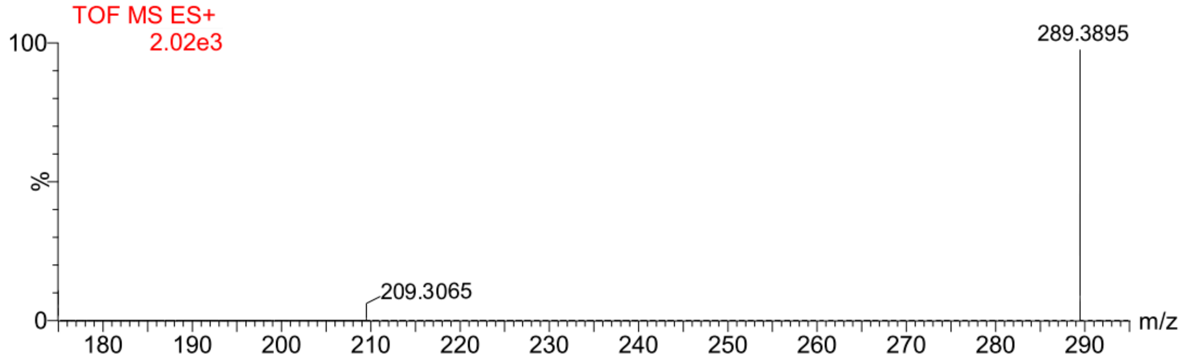

IR spectrum of compound 9

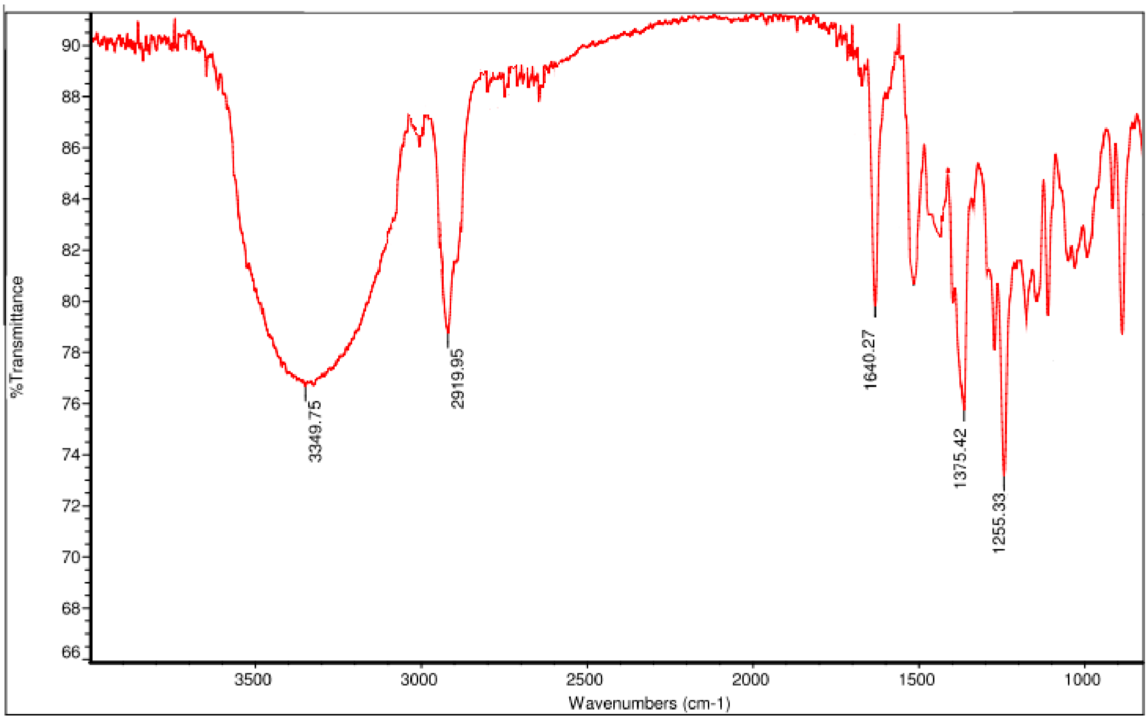

^{1}H NMR (400 MHz, $CDCl_3$) spectrum of compound 9

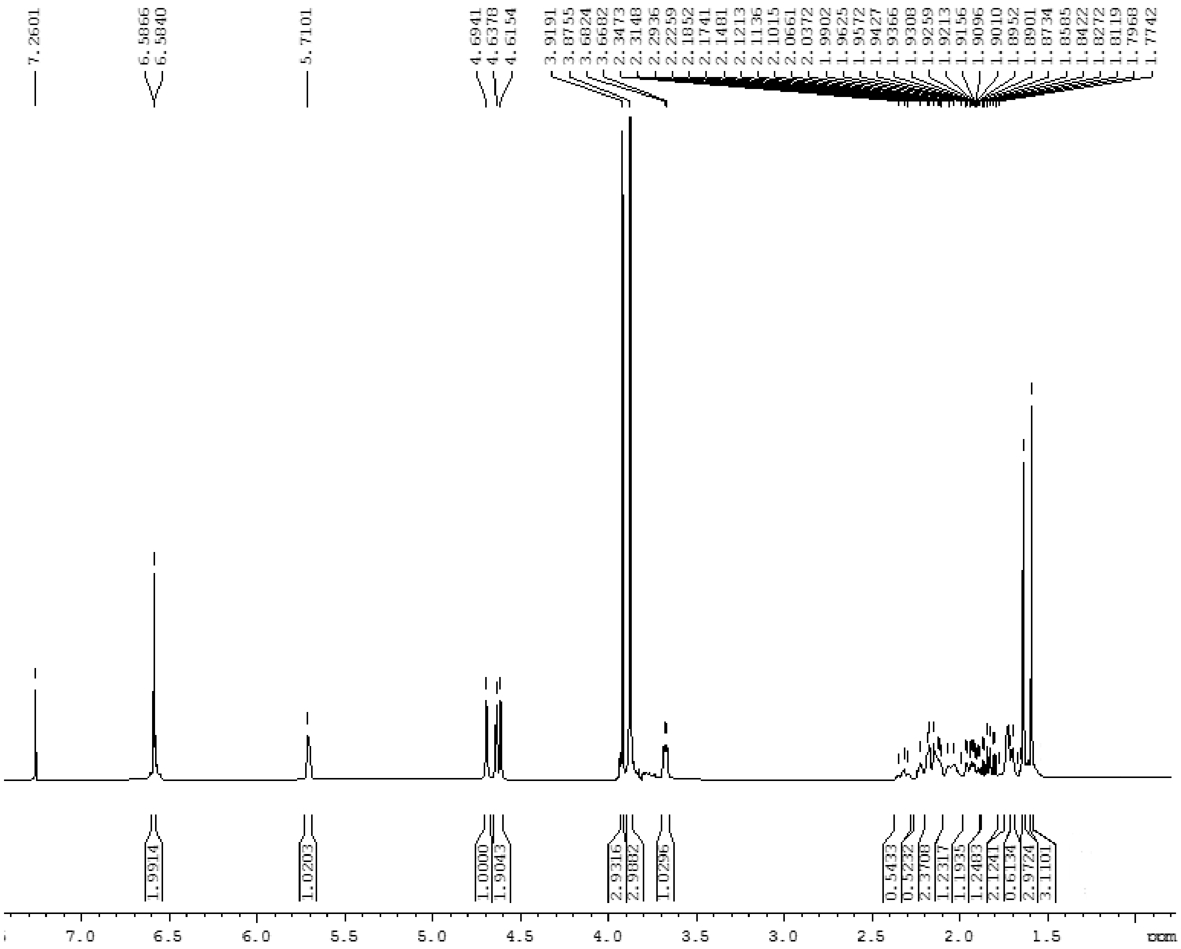

^{13}C NMR (100 MHz, $CDCl_3$) spectrum of compound 9

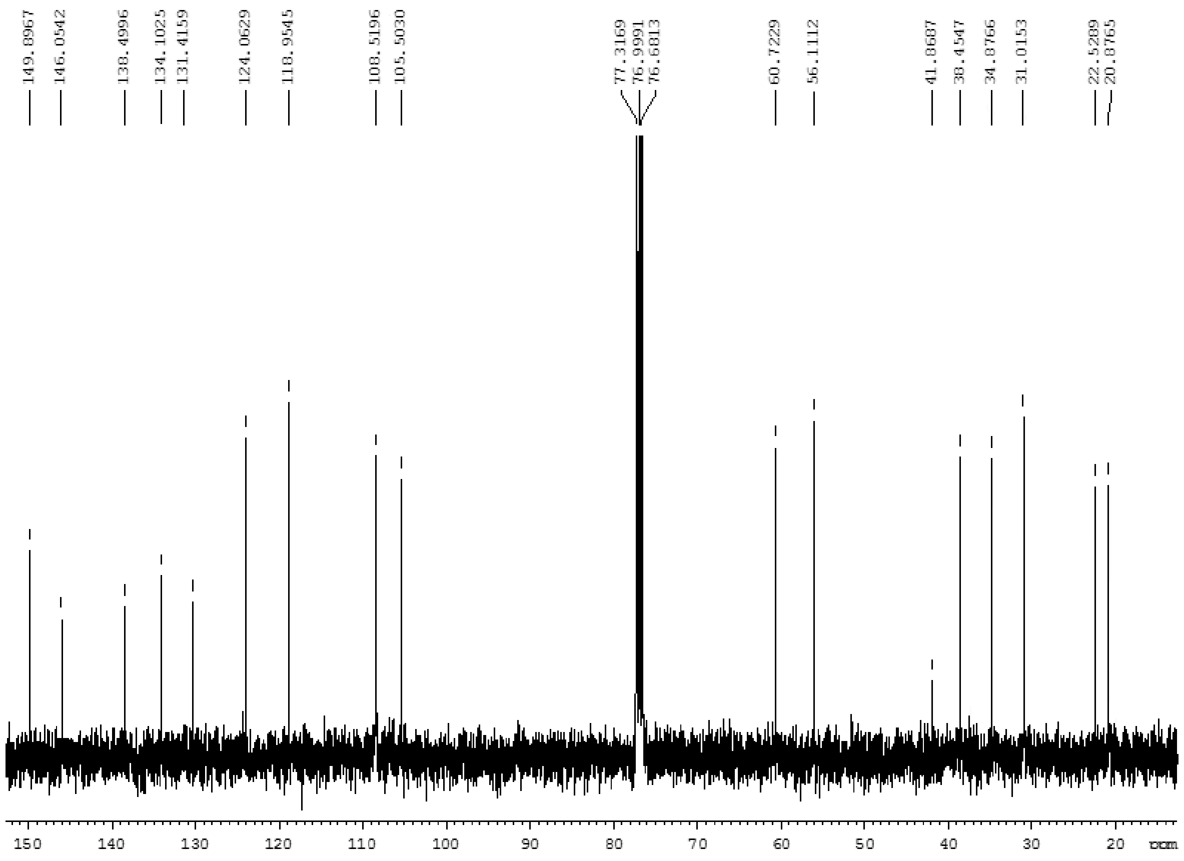

Mass of compound 9

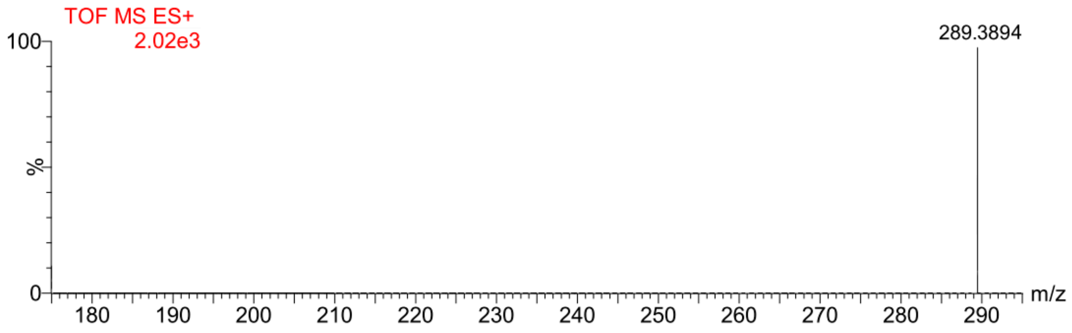

Tables

Table S1. Descriptors linked to the inhibition of *C. glabrata* obtained by multivariate analysis

Comp	Charge C_5	Charge C_6	Charge C_6^2	pMIC *C. glabrata* Obs	pMIC *C. glabrata* Calc	Res
**2**	-0.217	-0.335	0.112	4.183	4.395	-0.212
**3**	-0.31	-0.297	0.088	4.183	4.102	0.081
**4**	-0.224	-0.101	0.010	4.204	4.166	0.038
**5**	0.325	-0.300	0.090	5.108	5.243	-0.135
**6**	0.332	-0.377	0.142	5.721	5.556	0.165
**7**	-0.314	-0.011	0.000	4.249	4.282	-0.033
**8**	-0.242	-0.226	0.051	4.249	4.081	0.169
**9**	-0.102	-0.224	0.050	4.255	4.328	-0.074

Table S2. Proposed derivatives from compound **6** to improve the inhibitory activity of *C. glabrata*

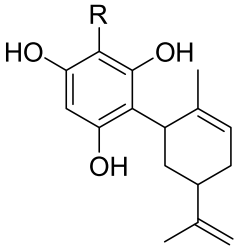

R	Charge C_5	Charge C_6	Charge C_6^2	pMIC *C. glabrata* Calc
**H**	0.332	-0.377	0.142	5.556
**F**	0.273	0.278	0.077	7.571
**Cl**	0.318	-0.172	0.03	5.055
**Br**	0.32	-0.255	0.065	5.126
**NO_2**	0.345	-0.072	0.005	5.247
**CHO**	0.363	-0.271	0.073	5.235
**COMe**	0.367	-0.267	0.071	5.234
**CO_2H**	0.365	-0.285	0.081	5.273
**Me**	0.329	-0.15	0.023	5.085
**Et**	0.319	-0.154	0.024	5.064
**NH_2**	0.31	0.005	0	5.464
**NMe_2**	0.307	0.012	0	5.492
**NHAc**	0.307	-0.012	0	5.385

Table S3. Descriptors linked to the inhibition of *C. lusitaniae* obtained by QSAR analysis

Comp.	$LUMO^2$	Charge C_1	Charge C_1^2	pMIC *C. lusitaniae* Obs	pMIC *C. lusitaniae* Calc	Res
**2**	$1.25x10^{-6}$	0.328	0.108	5.699	5.705	-0.006
**3**	$2.38x10^{-5}$	0.292	0.085	5.699	5.728	-0.029
**4**	$2.96\ x10^{-5}$	-0.068	0.005	5.745	5.772	-0.028
**5**	$1.82\ x10^{-5}$	0.009	0.000	5.721	5.682	0.039
**6**	$1.11\ x10^{-4}$	0.342	0.117	6.018	5.993	0.025
**7**	$1.30\ x10^{-5}$	-0.259	0.067	6.056	6.016	0.039
**8**	$6.45\ x10^{-5}$	-0.233	0.054	6.060	6.095	-0.035
**9**	$3.35\ x10^{-5}$	-0.259	0.067	6.060	6.067	-0.007

Table S4. Proposed derivatives from compound **7** to improve the inhibitory activity of *C. lusitaniae*

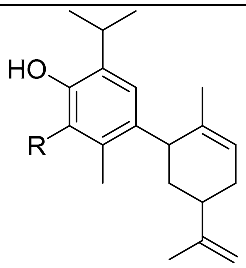

R	$LUMO^2$	C_1	C_1^2	pMIC *C. lusitaniae* Calc
**H**	0	-0.259	0.067	6.31
**F**	0	-0.25	0.063	5.97
**Cl**	0	-0.25	0.063	7.35
**Br**	0	-0.251	0.063	7.45
**NO_2**	0.009	-0.257	0.066	231
**CHO**	0.004	-0.261	0.068	116
**COMe**	0.004	-0.256	0.066	114
**CO_2H**	0.003	-0.26	0.068	88.5
**Me**	0	-0.252	0.064	7.94
**OH**	0	-0.252	0.064	6.12
**NH_2**	0	-0.254	0.065	6.40
**NMe_2**	0	-0.252	0.064	6.93
**NHAc**	0.067	-0.249	0.062	1678

Table S5. Descriptors linked to the inhibition of *C. guillermondii* obtained by multivariate analysis

Comp	L-H	Charge $C_{8,}$	Charge C_2^2	pMIC *C. guillermondii* Obs	pMIC *C. guillermondii* Calc	Res
**2**	0.212	0.005	0.108	5.699	5.734	-0.035
**3**	0.200	0.003	0.085	5.699	5.684	0.015
**4**	0.230	0.000	0.005	5.155	5.154	0.001
**5**	0.213	0.003	0.000	5.409	5.406	0.003
**6**	0.220	0.005	0.117	5.721	5.691	0.030
**7**	0.218	0.002	0.067	5.456	5.466	-0.010
**8**	0.216	0.002	0.054	5.456	5.451	0.004
**9**	0.218	0.002	0.067	5.456	5.464	-0.008

Table S6. proposed derivatives from compound **6** to improve the inhibitory activity of *C. guillermondii*

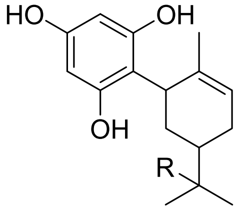

R	L-H	Charge $C_{7'}$	Charge C_2^2	pMIC *C. guillermondii* Calc
**H**	0.221	-0.276	0.016	-7.302
**F**	0.22	0.365	0.016	21.698
**Cl**	0.22	0.029	0.017	6.503
**Br**	0.213	0.026	0.017	6.417
**NO_2**	0.156	0.138	0.022	11.915
**CN**	0.221	-0.059	0.017	2.506
**CHO**	0.2	-0.104	0.017	0.638
**COMe**	0.198	-0.099	0.017	0.876
**CO_2H**	0.199	-0.087	0.016	1.413
**Me**	0.22	-0.057	0.016	2.608
**OH**	0.218	0.267	0.016	17.285
**OMe**	0.217	0.272	0.016	17.514
**NH_2**	0.204	0.114	0.016	10.47
**NMe_2**	0.199	0.136	0.016	11.502
**NHAc**	0.221	0.162	0.017	12.505

Table S7. Descriptors linked to the inhibition of *C. albicans* obtained by QSAR analysis

Comp	WI^2	MTI	MTI^2	pMIC *C. albicans* Obs	pMIC *C. albicans* Calc	Res
**2**	321489	4400	19360000	5.398	5.397	0.001
**3**	334084	4464	19927296	5.398	5.440	-0.042
**4**	425104	5226	27311076	5.409	5.497	-0.088
**5**	438244	5112	26132544	5.721	5.667	0.055
**6**	438244	4960	24601600	5.721	5.777	-0.056
**7**	1192464	8722	76073284	6.056	6.005	0.050
**8**	462400	5412	29289744	5.770	5.583	0.187
**9**	891136	7596	57699216	5.745	5.851	-0.106

Tabla S8. Proposed derivatives from compound **7** to improve the inhibitory activity of *C. albicans*

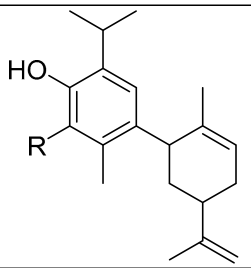

R	WI^2	MTI	MTI^2	pMIC *C. albicans* Calc
**Me**	1456849	9624	92621376	6.149
**Et**	1784896	10656	113550336	6.106
***i*Prop**	2152089	11704	136983616	5.993
***t*But**	2560000	12768	163021824	5.807
**OH**	1456849	9412	88585744	6.548
**OMe**	1784896	10440	108993600	6.57
**OEt**	2220100	11666	136095556	6.513
**O-*i*Prop**	2709316	12908	166616464	6.362
**O-*t*But**	3254416	14166	200675556	6.111
**OCF_3**	3254416	12735	162180225	10.264
**OAc**	2709316	12594	158608836	7.216
**OMOM**	2788900	12782	163379524	7.208
**OBz**	6310144	19784	391406656	3.879
